# Biological activity of mistletoe: in vitro and in vivo studies and mechanisms of action

**DOI:** 10.1007/s12272-020-01247-w

**Published:** 2020-07-03

**Authors:** Anna Szurpnicka, Anna Kowalczuk, Arkadiusz Szterk

**Affiliations:** 1 Department of Natural Medicinal Products and Dietary Supplements, National Medicines Institute, Chełmska 30/34, 00-725 Warsaw, Poland; 2 National Medicines Institute, Chełmska 30/34, 00-725 Warsaw, Poland; 3 Department of Spectrometric Methods, National Medicines Institute, Chełmska 30/34, 00-725 Warsaw, Poland

**Keywords:** Mistletoe, *Viscum*, Extracts, Bioactivities, Mechanisms, Pharmacognosy

## Abstract

Mistletoe has been used as treatment of many diseases in traditional and folk medicine. To date, anticancer, immunomodulatory, cardiac, antidiabetic, hepatoprotective, neuropharmacological, antibacterial and antifungal properties of mistletoe extracts have been studied the most. In this review, we summarized in vitro and in vivo studies on the pharmacological activity of *Viscum* species. Furthermore, we proposed the possible mechanisms of action of this herb, which might include many signalling pathways. Mistletoe could regulate either similar or different targets in various pathways that act on membrane receptors, enzymes, ion channels, transporter proteins and transcriptional targets. Still, pharmacological activities of mistletoe have been investigated mainly for crude extracts. It is a new field for scientists to determined which chemical compounds are responsible for the individual biological activities of mistletoe and how these activities are achieved. As a result, mistletoe might become a source of new complementary therapies supporting the treatment of many diseases.

## Introduction

Mistletoe (*Viscum* L.) belongs to the family of Viscaceae. In Europe, Asia, Africa and Australia, about 100 species of mistletoe can be distinguished, of which the most known are in Santalaceae: *Viscum album* L. (European mistletoe), Santalaceae: *Viscum album* subsp. *Coloratum* Kom. (*Viscum coloratum* (Kom.) Nakai, Korean mistletoe), Santalaceae: *Viscum articulatum* Burm. f., Santalaceae: *Viscum shimperi* Engl., Santalaceae: *Viscum capense* L.f. and Santalaceae: *Viscum cruciatum* Sieber ex Boiss. Mistletoe is a semi-parasitic evergreen shrub, which means it depends on having water and some nutrients supplied from another plant (host tree) while it produces carbohydrates in a process of photosynthesis. *Viscum* species inhabit many types of wooded habitats and parasitize both deciduous and coniferous trees (Bussing 2000). For clinical applications, the most popular species are mistletoe parasitizing fir, maple, almond, birch, hawthorn, ash, apple, pine, poplar, oak, willow, lime and elm (Kienle et al. 2011). *Viscum* species have been used in the traditional medicine of Europe for centuries. Hippocrates used mistletoe to treat diseases of the spleen and complaints associated with menstruation, while Pliny the Elder used it to treat epilepsy, infertility and ulcers. In the Middle Ages, Paracelsus recommended mistletoe as a treatment for epilepsy. Hildegard von Bingen described mistletoe as a treatment for diseases of the spleen and liver. Mistletoe was also applied for deworming children, to treat labour pains, gout, affections of the lungs and liver, leprosy, mumps, fractures and hepatitis. During the eighteenth century, mistletoe was applied for “weakness of the heart” and oedema (Bussing 2000). By the end of the nineteenth century, mistletoe was rejected by scientists as a folklore remedy. The scientific interest on mistletoe was awakened in the twentieth century, as Gaultier investigated the effect of oral or subcutaneous applications of fresh *Viscum album* L. extracts on blood pressure in humans and animals (Bussing 2000; Committee on Herbal Medicinal Products 2012). In 1920, *Viscum album* L. was introduced as a cancer treatment by Rudolf Steiner who recommended a drug extract produced in a complicated manufacturing process combining sap from mistletoe harvested in the winter and summer (Bussing 2000). Mistletoe was also commonly used in other parts of the world. In Japan, mistletoe was used to treat hypertension, spasms of the heart, rheumatic pain, threatened abortion and locally to treat frostbite. In India, a tea prepared from mistletoe leaves was used to treat diabetes, while a preparation of *Viscum articulatum* Burm. f. was given in fevers with aching limbs. In Africa, *Viscum* species were a remedy to treat diarrhoea and an enema for stomach troubles in children. In Israel, *Viscum cruciatum* Sieber ex Boiss. was commonly used to treat constipation in young children and adults. Mistletoe was also used against general pain, backache and arthritis. In the traditional medicine of Egypt, the plant was used for the treatment of epilepsy, arteriosclerosis, and diseases of cardiac arteries, and as a hypotensive (Bussing 2000; Lev et al. 2011; Committee on Herbal Medicinal Products 2012). Such varied pharmaceutical applications result from the rich chemical composition of *Viscum* species, which largely depend on the host species. The main active compounds are lectins, viscotoxins, flavonoids, phenolic acids, sterols, lignans, terpenoids, phenylpropanoids, alkaloids and fatty acids (Szurpnicka et al. 2019). In this review, we would like to summarize the scientific data on the pharmacological activity of *Viscum* species and analyse the probable mechanisms of actions of mistletoe.

## Anticancer and immunomodulatory activity

**Fig. 1:**
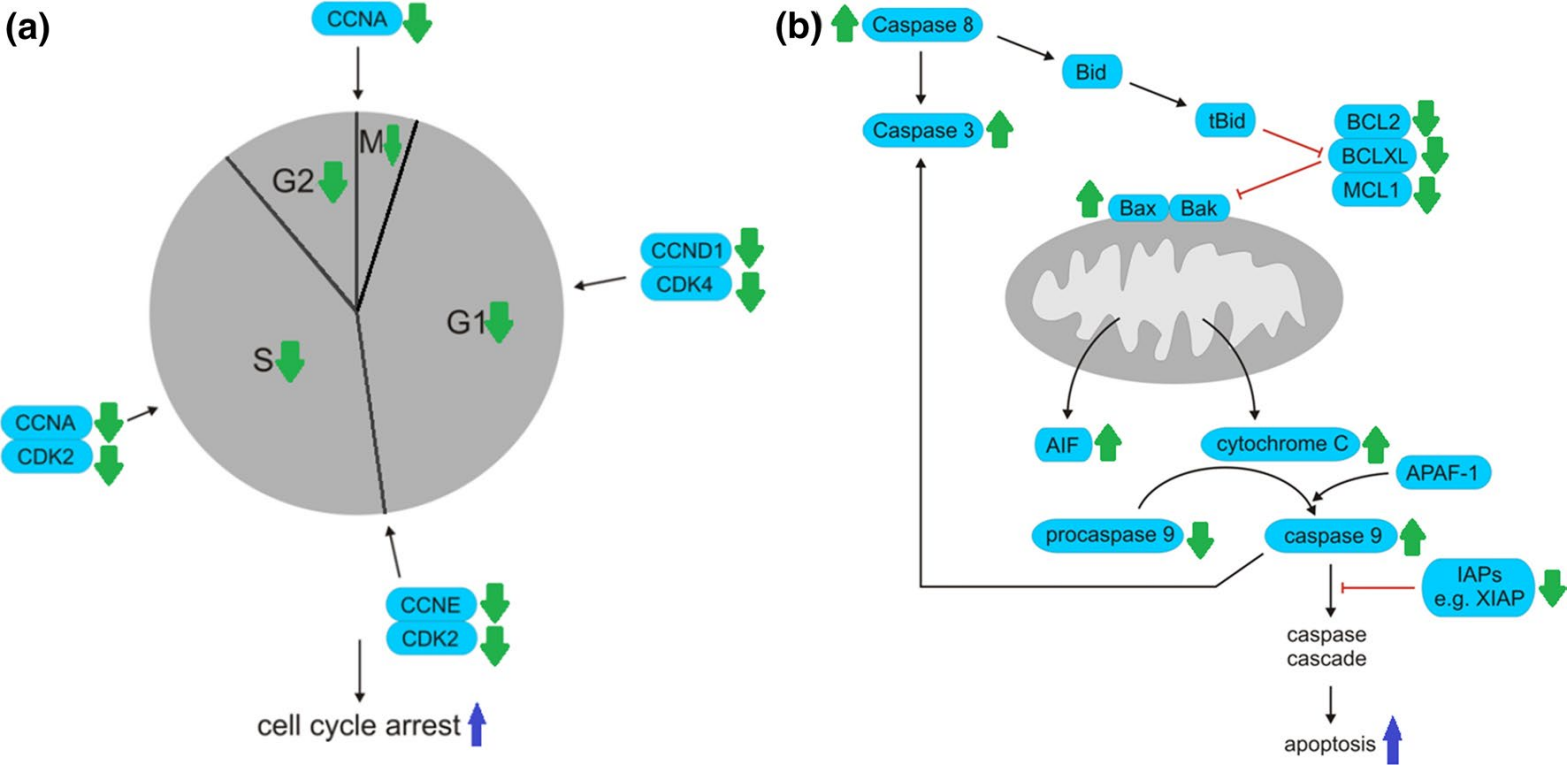
Mechanism of anticancer activity of mistletoe. Mistletoe targets two important signalling pathways, PI3K/AKT and MAPK. PI3K/AKT pathway is responsible for growth and survival of cancer cells. Mistletoe induces apoptosis by inhibition of AKT phosphorylation. MAPK pathway is mediated by ERK, p38 and JNK. Mistletoe enhances p38 and JNK1 activation and reduces ERK leading to apoptosis and cell cycle arrest of cancer cells. **a** Mistletoe downregulates cyclins (CCND1, CCNE, CCNA) and cyclin-dependent protein kinases (CDK4, CDK2) inhibiting cell cycle. **b** Mistletoe upregulates proapoptotic proteins (Bax) and downregulates inhibitors of apoptosis (IAPs) such as BCL2, BCL2L1, MCL1, XIAP. Furthermore, mistletoe leads to release of cytochrome c and activation of caspases resulting in apoptosis

In German-speaking countries, mistletoe has been used as complementary anticancer therapy for more than 100 years. *Viscum album* L. preparations can be divided into phytotherapeutic extracts standardized on a certain lectin level (brand names such as Cefalektin, Eurixor, Lektinol) and anthroposophical/homeopathically produced extracts (brand names such as Abnoba*Viscum*, Helixor, Iscador, Iscucin, Isorel) (Freuding et al. 2019). The main anticancer compounds isolated from *Viscum* species are lectins (Thies et al. 2005; Eggenschwiler et al. 2007) and viscotoxins (Schaller et al. 1996). Later studies have shown that other compounds, such as phenolic compounds (Melo et al. 2018), triterpene acids (Delebinski et al. 2015) and non-polar compounds (Ćebović et al. 2008), have also shown antitumor properties. Furthermore, it was reported that complete mistletoe extract is more potent at inhibiting tumour cells than isolated compounds (Felenda et al. 2019), and there is synergistic action between different groups of mistletoe compounds (Twardziok et al. 2016; Kleinsimon et al. 2017). Mistletoe shows bi-directional activity in the treatment of cancer. Firstly, it affects the quality of life of cancer patients by the improvement of fatigue, sleep, exhaustion, nausea, vomiting, appetite, depression, anxiety, pain and side effects of traditional treatment (Kienle and Kiene 2010; Brandenberger et al. 2012; Kim et al. 2012). Secondly, it shows antitumor activity by cytotoxicity, induction of apoptosis (Ćebović et al. 2008; Park et al. 2012; Han et al. 2015; Mishra et al. 2018) and inhibition of angiogenesis (Park et al. 2001; Elluru et al. 2009). The mechanism of action is shown in Fig. 1. In vitro studies on anticancer activity of mistletoe have confirmed that it modulates many different pathways, playing key roles in tumour proliferation, including MAPK (mitogen-activated protein kinase) (Park et al. 2012) and PI3K/AKT (phosphatidylinositol 3-kinase/protein kinase B) (Fan et al. 2019). Furthermore, mistletoe can cause cell cycle arrest (Dela Cruz et al. 2015; Kim et al. 2017; Melo et al. 2018), loss of mitochondrial membrane permeability (MMP) (Mishra et al. 2018) and can activate caspases and regulate pro- and anti-apoptotic proteins (Fan et al. 2019) (Table 1). The anticancer activity of *Viscum* species is linked with their immunomodulatory activity (Oei et al. 2019), such as the increase of maturation and activation of dendritic cells (Elluru et al. 2008; Kim et al. 2014a; Steinborn et al. 2017), abrogation of tumour-induced immunosuppression of dendritic cells (Steinborn et al. 2017), increase of leukocytes, eosinophils, granulocytes (Huber et al. 2005, 2011) and lymphocytes (Semiglasov et al. 2004), increase of cytokines secretion (Hajto et al. 1990; Kovacs 2000; Elluru et al. 2008), increase of activity of natural killer cells (Hajto 1986; Tabiasco et al. 2002; Braedel-Ruoff 2010; Kim et al. 2018), increase of the activities of natural killer cells during surgery (Schink et al. 2007) and enhancement of cellular and humoral immune response (Yoon et al. 2001; Gardin 2009). Clinical studies were done on patients suffering from cancer diseases such as bladder cancer, breast cancer, colorectal cancer, glioma, lung cancer, melanoma and the results of these studies have been published in many articles. Those who are interested in the topic are invited to read review articles focusing on the anti-cancer properties of mistletoe (Ernst et al. 2003; Bar-Sela 2011; Bar-Sela et al. 2013; Steele et al. 2015; Kienle et al. 2016; Schläppi et al. 2017; Freuding et al. 2019). Additionally, it is worth paying attention to studies regarding synergistic interactions of mistletoe preparations with other cancer treatments such as chemotherapy and radiotherapy (Siegle et al. 2001; Hong et al. 2014; Kleinsimon et al. 2017; Schötterl et al. 2019; Menke et al. 2019). Furthermore, we found research that Korean mistletoe lectin affected the self-renewal activity of placenta-derived mesenchymal stem cells (MSCs) (Choi et al. 2012; Kim et al. 2019), however its therapeutic use for cancer is still insufficiently investigated (Hmadcha et al. 2020).

**Table 1:** Antitumor activity of *Viscum* species—in vitro studies

Mechanism of action	Preparation/compound (host tree)	Concentration of the extract/compound	Cell line	Observations	References
Cell cycle	*Viscum album* L. (apple tree)	Extract containing 10 ng/mL lectin MLI Extract containing 60 µg/mL oleanolic acid Extract containing 5 ng/mL lectin MLI and 50 µg/mL oleanolic acid	Human osteosarcoma cell lines 143B, Saos-2 and U2OS	G1 arrest in TP53 wild-type (U2OS) and null-mutant (Saos-2) cells, S arrest in TP53 mutant cells (143B), blockage of G1/S transition accompanied by downregulation of CDK4, CCND1, CDK2, CCNE, CCNA, investigations on the transcriptional level revealed secondary TP53 participation, cell cycle arrest was mediated by transcriptionally increased expression of GADD45A and CDKN1A and decreased SKP2 levels	Kleinsimon et al. (2018)
	*Viscum album* L	3% and 5% v/v	Murine melanoma cell line B16F10	Increased Sub G0 population, probably associated with a consistent decrease in G1, and an increase in S or G2/M populations	Melo et al. (2018)
	*Viscum articulatum* Burm. f. (*Dalbergia latifolia* Roxb*.*)	0.015–150 µg/mL	Human leukemia cell lines Jurkat E6.1 and THP1	G2/M cell cycle arrest with a concomitant decrease in some cells at G0/G1 phase	Mishra et al. (2018)
	1,7-Bis(4-hydroxyphenyl)-1,4-heptadien-3-one isolated from *Viscum coloratum* (Kom.) Nakai	2.5–20 µM	Human lung cancer cell lines A549 and NCI-H292	Cell cycle arrest in A549 and NCI- H292 cells at the S and G0/G1 phases, respectively	Fan et al. (2019)
	*Viscum coloratum* (Kom.) Nakai	Lectin 10–1000 ng/mL Extract 10–1000 µg/mL	Mouse melanoma cell lines B16BL6 and B16F10	G0/G1 cell cycle arrest	Han et al. (2015)
MMP	*Viscum articulatum* Burm. f. (*Dalbergia latifolia* Roxb*.*)	0.015–150 µg/mL	Human leukemia cell lines Jurkat E6.1 and THP1	Loss of MMP, which is required for cytochrome c release	Mishra et al. (2018)
	*Viscum album* L (apple tree)	Extract containing 1.25–7.5 ng/mL lectin MLI Extract containing 30–45 µg/mL oleanolic acid Extract containing 1.25–7.5 ng/mL lectin MLI and 30–45 µg/mL oleanolic acid	Human alveolar Rhabdomyosarcoma cell line RMS-13		Stammer et al. (2017)
	*Viscum album* L (apple tree)	Extract containing 1–40 ng/mL lectin MLI Extract containing 10–60 µg/mL oleanolic acid Extract containing 1–40 ng/mL lectin MLI and 10–60 µg/mL oleanolic acid	Human Ewing sarcoma cell lines TC-71 and MHH-ES-1		Twardziok et al. (2016)
	*Viscum album* L. (apple tree)	Extract containing 2–16 ng/mL lectin MLI Extract containing 20–40 µg/mL oleanolic acid Extract containing 2–16 ng/mL lectin MLI and 20–40 µg/mL oleanolic acid	Human acute myeloid leukemia cell lines U937 and HL-60		Delebinski et al. (2015)
	1,7-Bis(4-hydroxyphenyl)-1,4-heptadien-3-one isolated from *Viscum coloratum* (Kom.) Nakai	2.5–20 µM	Human lung cancer cell lines A549 and NCI-H292		Fan et al. (2019)
	*Viscum album* L. (apple tree)	Extract containing 2.5–10 ng/mL lectin MLI Extract containing 40–60 µg/mL oleanolic acid Extract containing 2.5–10 ng/mL lectin MLI and 40–60 µg/mL oleanolic acid	Human osteosarcoma cell lines 143B and Saos-2		Kleinsimon et al. (2017)
Cytochrome *c* and AIF	*Viscum album* L. (apple tree)	Extract containing 4–8 ng/mL lectin MLI Extract containing 25–35 µg/mL oleanolic acid Extract containing 4–8 ng/mL lectin MLI and 25–35 µg/mL oleanolic acid	Human acute myeloid leukemia cell lines U937 and HL-60	Release of cytochrome c	Delebinski et al. (2015)
	1,7-Bis(4-hydroxyphenyl)-1,4-heptadien-3-one isolated from *Viscum coloratum* (Kom.) Nakai	2.5–20 µM	Human lung cancer cell lines A549 and NCI-H292	Release of cytochrome c and AIF	Fan et al. (2019)
MAPK/JNK, ERK and p38	Lectin II isolated from *Viscum coloratum* (Kom.) Nakai	100 ng/mL	Human myeloid leukemia cell line U937	Increased phosphorylation of the JNK1 substrate, GST-c-Jun N-terminal protein	Park et al. (2000)
	Abnoba*Viscum* F (ash)	20 µg/mL	Human myeloid leukemia cell line K562	Increased phosphorylation of JNK1 and p38, reduced levels of phosphorylated ERK-1/2	Park et al. (2012)
	*Viscum album* L. (apple tree)	Extract containing 1–40 ng/mL lectin MLI Extract containing 10–60 µg/mL oleanolic acid Extract containing 1–40 ng/mL lectin MLI and 10–60 µg/mL oleanolic acid	Human Ewing sarcoma cell lines TC-71 and MHH-ES-1	Increased phosphorylation of JNK1 and p38	Twardziok et al. (2017)
	*Viscum album* L. (apple tree)	Extract containing 10 ng/mL lectin MLI Extract containing 60 µg/mL oleanolic acid Extract containing 5 ng/mL lectin MLI and 50 µg/mL oleanolic acid	Human osteosarcoma cell lines 143B, Saos-2 and U2OS	Activation of JNK1 with simultaneous inactivation of ERK-1/2	Kleinsimon et al. (2018)
	1,7-Bis(4-hydroxyphenyl)-1,4-heptadien-3-one isolated from *Viscum coloratum* (Kom.) Nakai	2.5–20 µM	Human lung cancer cell lines A549 and NCI-H292	Upregulation of the expression levels of p-ERK1/2 and p-P90RSK	Fan et al. (2019)
PI3K/AKT	1,7-Bis(4-hydroxyphenyl)-1,4-heptadien-3-one isolated from *Viscum coloratum* (Kom.) Nakai	2.5–20 µM	Human lung cancer cell lines A549 and NCI-H292	Downregulation of the phosphorylation of AKT and P70RSK	Fan et al. (2019)
	Lectin isolated from *Viscum coloratum* (Kom.) Nakai	10 ng/mL	Human cancer cell line A253	Dephosphorylation of AKT	Choi et al. (2004)
	Iscador Qu Spezial (oak) Iscador M (apple tree)	0.3 mg/mL	Human tongue cancer cell lines SCC9 and SCC25	Reduced pAKT	Klingbeil et al. (2013)
	Abnoba*Viscum* F (ash)	20 µg/mL	Human myeloid leukemia cell line K562	Reduced levels of phosphorylated AKT	Park et al. (2012)
COX-2	VA Qu Spez (oak)	10–100 µg/mL	Human lung adenocarcinoma cell line A549	Inhibition of the secretion of IL-1β-induced PGE2 associated with a reduced expression of COX-2	Hegde et al. (2011) and Saha et al. (2015)
Caspases	*Viscum album* L. (apple tree)	Extract containing 2–16 ng/mL lectin MLI Extract containing 20–40 µg/mL oleanolic acid Extract containing 2–16 ng/mL lectin MLI and 20–40 µg/mL oleanolic acid	Human acute myeloid leukemia cell line HL-60	Activation of caspase-8 and caspase -9	Delebinski et al. (2015)
	*Viscum articulatum* Burm. f. (*Dalbergia latifolia Roxb.*)	0.015–150 µg/mL	Human leukemia cell lines Jurkat E6.1 and THP1	Activation of caspase-8 and caspase-3	Mishra et al. (2018)
	*Viscum album* L. (apple tree)	Extract containing 1.25–7.5 ng/mL lectin MLI Extract containing 30–45 µg/mL oleanolic acid Extract containing 1.25–7.5 ng/mL lectin MLI and 30–45 µg/mL oleanolic acid	Human alveolar Rhabdomyosarcoma cell line RMS-13	Activation of caspase-9, caspase-8 and caspase-3	Stammer et al. (2017)
	*Viscum album* L. (apple tree)	Extract containing 1–40 ng/mL lectin MLI Extract containing 10–60 µg/mL oleanolic acid Extract containing 1–40 ng/mL lectin MLI and 10–60 µg/mL oleanolic acid	Human Ewing sarcoma cell lines TC-71 and MHH-ES-1	Activation of caspase-9, caspase-8	Twardziok et al. (2016)
	1,7-Bis(4-hydroxyphenyl)-1,4-heptadien-3-one isolated from *Viscum coloratum* (Kom.) Nakai	2.5–20 µM	Human lung cancer cell lines A549 and NCI-H292	Activation of caspase-9 and caspase-3	Fan et al. (2019)
	Abnoba*Viscum* F (ash)	20 µg/mL	Human myeloid leukemia cell line K562	Decreased expression of procaspase-9 but increased that of cleaved (active) caspase-9	Park et al. (2012)
	Lectin isolated from *Viscum coloratum* (Kom.) Nakai	10 ng/mL	Human cancer cell line A253	Activation of caspase-3	Choi et al. (2004)
	*Viscum album* L. (apple tree)	Extract containing 2.5–10 ng/mL lectin MLI Extract containing 40–60 µg/mL oleanolic acid Extract containing 2.5–10 ng/mL lectin MLI and 40–60 µg/mL oleanolic acid	Human osteosarcoma cell lines 143B and Saos-2	Activation of caspase-8 and caspase-9	Kleinsimon et al. (2017)
	*Viscum Coloratum* (Kom.) Nakai	Lectin 10–1000 ng/mL Extract 10–1000 µg/mL	Mouse melanoma cell lines B16BL6 and B16F10	Activation of caspase-1, 3, 4, 5, 6, 7, 8, and 9	Han et al. (2015)
Antiapoptotic proteins	*Viscum album* L. (apple tree)	Extract containing 40 ng/mL lectin MLI Extract containing 40 µg/mL oleanolic acid Extract containing 15 ng/mL lectin MLI and 30 µg/mL oleanolic acid	Human acute myeloid leukemia cell line HL-60	Downregulation of BIRC5, XIAP, p53 and claspin	Delebinski et al. (2015)
	*Viscum album* L. (apple tree)	Extract containing 1–40 ng/mL lectin MLI Extract containing 10–60 µg/mL oleanolic acid Extract containing 1–40 ng/mL lectin MLI and 10–60 µg/mL oleanolic acid	Human Ewing sarcoma cell lines TC-71 and MHH-ES-1	Downregulation of BIRC5, XIAP, MCL1 and CLSPN,	Twardziok et al. (2016)
	*Viscum album* L. (apple tree)	Extract containing 2.5–10 ng/mL lectin MLI Extract containing 40–60 µg/mL oleanolic acid Extract containing 2.5–10 ng/mL lectin MLI and 40–60 µg/mL oleanolic acid	Human osteosarcoma cell lines 143B and Saos-2	Downregulation of BIRC5, XIAP, BCL2, and CLSPN	Kleinsimon et al. (2017)
	*Viscum album* L. (apple tree)	Extract containing 5 ng/mL lectin MLI Extract containing 40 µg/mL oleanolic acid Extract containing 5 ng/mL lectin MLI and 40 µg/mL oleanolic acid	Human alveolar Rhabdomyosarcoma cell lines RH-30 and RMS-13	Downregulation of BIRC5, XIAP, BCL2, BCL2L1 and MCL1	Stammer et al. (2017)
	*Viscum articulatum* Burm. f. (*Dalbergia latifolia Roxb.*)	0.015–150 µg/mL	Human leukemia cell lines Jurkat E6.1 and THP1	Downregulation of BCL2	Mishra et al. (2018)
	Lectin isolated from *Viscum coloratum* (Kom.) Nakai	10 ng/mL	Human hepatocarcinoma cells SK-Hep-1 and Hep3B		Lyu et al. (2002)
	Abnoba*Viscum* F (ash)	20 µg/mL	Human myeloid leukemia cell line K562	Downregulation of Mcl-1	Park et al. (2012)
	1,7-bis(4-hydroxyphenyl)-1,4-heptadien-3-one isolated from *Viscum coloratum* (Kom.) Nakai	2.5–20 µM	Human lung cancer cell lines A549 and NCI-H292	Upregulation of Bcl-2 and Bcl-xL	Fan et al. (2019)
Proapoptotic proteins	*Viscum articulatum* Burm. f. (*Dalbergia latifolia Roxb.*)	0.015–150 µg/mL	Human leukemia cell lines Jurkat E6.1 and THP1	Upregulation of Bax	Mishra et al. (2018)
	Lectin isolated from *Viscum coloratum* (Kom.) Nakai	10 ng/mL	Human hepatocarcinoma cells SK-Hep-1 and Hep3B		Lyu et al. (2002)
	*Viscum album* L. (apple tree)	Extract containing 2.5–10 ng/mL lectin MLI Extract containing 40–60 µg/mL oleanolic acid Extract containing 2.5–10 ng/mL lectin MLI and 40–60 µg/mL oleanolic acid	Human osteosarcoma cell lines 143B and Saos-2		Kleinsimon et al. (2017)
	1,7-Bis(4-hydroxyphenyl)-1,4-heptadien-3-one isolated from *Viscum coloratum* (Kom.) Nakai	2.5–20 µM	Human lung cancer cell lines A549 and NCI-H292	Downregulation of Bax	Fan et al. (2019)
STAT3	*Viscum album* L. (apple tree)	Extract containing 10 ng/mL lectin MLI Extract containing 60 µg/mL oleanolic acid Extract containing 5 ng/mL lectin MLI and 50 µg/mL oleanolic acid	Human osteosarcoma cell lines 143B, Saos-2 and U2OS	Dephosphorylation of STAT3 at Tyr705 and Ser727, down-regulation of total STAT3 and its direct downstream targets BIRC5 and C-MYC	Kleinsimon et al. (2018)
	Abnoba*Viscum* F (ash)	5–20 µg/mL	Human hepatocellular carcinoma cell line Hep3B	Reduction of C-MYC protein levels which might be mediated by the ubiquitin–proteasome system	Yang et al. (2019)
Telomerase	Lectin isolated from *Viscum coloratum* (Kom.) Nakai	10 ng/mL	Human cancer cell line A253	Inhibition of telomerase activity through downregulation of hTERT	Choi et al. (2004)
ROS	*Viscum articulatum* Burm. f. (*Dalbergia latifolia Roxb.*)	0.015–150 µg/mL	Human leukemia cell lines Jurkat E6.1 and THP1	ROS mediated DNA fragmentation	Mishra et al. (2018)
	1,7-Bis(4-hydroxyphenyl)-1,4-heptadien-3-one isolated from *Viscum coloratum* (Kom.) Nakai	5–20 µM	Human lung cancer cell lines A549 and NCI-H292	Promotion of ROS generation	Fan et al. (2019)

## Cardiac activity

**Fig. 2:**
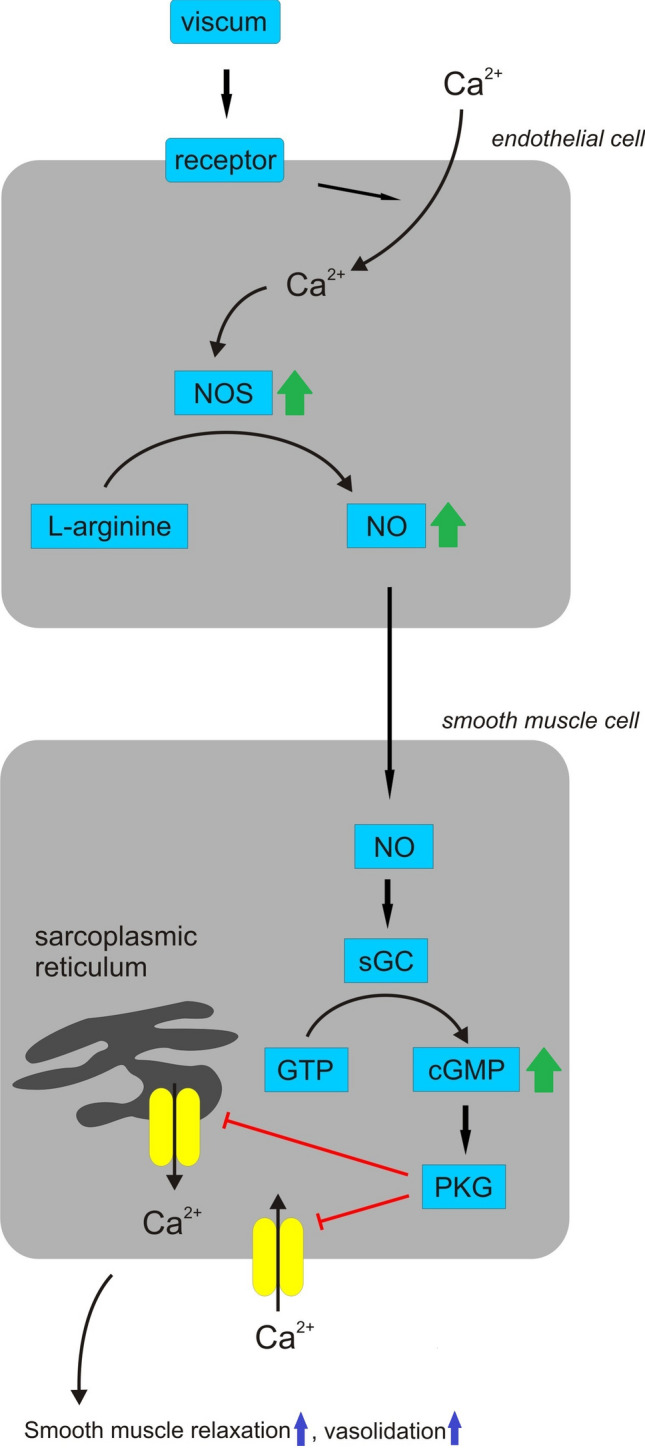
Mechanism of cardiac activity of mistletoe. Mistletoe compounds acting on receptor of endothelial cell might activate influx of Ca^2+^ ions leading to activation of NOS. NOS catalyzes formation of NO from L-arginine. NO diffuses to smooth muscle cell. Once sGC is activated by NO, GTP to cGMP conversion is triggered. cGMP activates PKG leading to reducing intracellular Ca^2+^ (by inhibition of Ca^2+^ influx through ligand gated Ca^2+^ channels and release from cellular stores). Proposed mechanism is confirmed by the fact that mistletoe induces NOS-2 and NOS-3 overexpression which is connected with increase in NO and cGMP production

The cardiac activity of mistletoe has been confirmed in in vitro as well as in vivo studies (Committee on Herbal Medicinal Products 2012; Poruthukaren et al. 2014; Montero et al. 2016) (Table 2). Tenorio et al. (2005) and Tenorio-Lopez et al. (2006) studied the mechanism of vasodilator activity of aqueous extract of *Viscum album* L. leaves on the Langendorff’s isolated and perfused heart model. They showed that mistletoe induced nitric oxide syntetaze-2 (NOS-2) and nitric oxide syntetaze-3 (NOS-3) overexpression, which was connected with increases in nitric oxide (NO) and cyclic guanosine monophosphate (cGMP) production. Therefore, the vasodilator activity of mistletoe might be mediated by the NO/sGC pathway. Soluble guanylyl cyclase (sGC) is an enzyme catalysing the conversion of GTP to cGMP and mediating several biological functions, such as the inhibition of platelet aggregation, smooth muscle relaxation and vasodilation. NO activates sGC by directly binding to heme to form a ferrous–nitrosyl–heme complex. Once sGC is activated by NO, GTP to cGMP conversion is triggered. Exogenous and endogenous compounds produce vasodilation through increases in cGMP, which in turn, relaxes vascular smooth muscle cells by both desensitising the contractile apparatus to Ca^2+^ and lowering intracellular Ca^2+^, with the consequent activation of a protein known as cGMP-dependent protein kinase. NO, synthesised by the enzyme nitric oxide synthase (NOS), maintains a vasodilator tone that is essential for the regulation of blood flow and pressure. NOS is a heme-containing enzyme that has three isoforms, designated as NOS-1, NOS-2 and NOS-3 (Tenorio-Lopez et al. 2006). Furthermore, studies on myocardial ischemia and reperfusion injury in rats as well as isoproterenol-induced heart failure in rats confirmed the cardioprotective effect of *Viscum album* L. might be mediated by the upregulation of the NO pathway (Karagöz et al. 2016; Suveren et al. 2017). Studies of Nω-nitro-L-arginine methyl ester (L-NAME)-induced hypertensive rats treated with methanolic extract of *Viscum articulatum* Burm. f. showed that mistletoe has an antihypertensive effect, which may be attributed to its diuretic, nephroprotective and hypolipidemic action. It was proposed the blood pressure lowering activity of this extract might have been due to the presence of triterpenoids, such as oleanolic acid and betulinic acid (Bachhav et al. 2012). Oleanolic acid isolated from the cuticular wax of *Viscum articulatum* Burm. f. significantly decreased the systolic blood pressures and cardiac lipid peroxidation levels in glucocorticoid (dexamethasone)-induced hypertensive rats, which might be connected with its antioxidant and nitric oxide releasing action (Bachhav et al. 2011). On the other hand, a study carried out in L-NAME-induced hypertensive rats treated with oleanolic acid showed that this compound did not affect nitric oxide levels, and its antihypertensive effect might be due to diuresis and nephroprotection (Bachhav et al. 2015). Another mechanism of the antihypertension activity of *Viscum* might be mediated by the calcium channel blockade (CCB). The contractile mechanism in smooth muscle is activated by a rise in the concentration of free intracellural Ca^2+^ concentration, which activates the contractile elements. The increase in intracellular Ca^2+^ occurs via either influx from the extracellular fluid through voltage-dependant Ca^2+^ channels (VDCs) or its release from intracellular stores. Thus, vascular smooth muscle relaxant agents may produce their effects by inhibiting either or both sources of Ca^2+^ (Mojiminiyi et al. 2008; Khan et al. 2016). A study carried out in rat aortic rings showed that an aqueous extract of leaves of *Viscum album* L. growing on oil palm trees had vasorelaxant activity, which might be mediated by a non-specific non-competitive inhibition of Ca^2+^ influx as well as inhibition of Ca^2+^ mobilization from intracellular stores (Mojiminiyi et al. 2008). Furthermore, a study carried out in rabbit aortic rings showed that vasorelaxant activity of mistletoe is mediated through a voltage-dependent Ca^2+^ channel blockade (Khan et al. 2016). It was proposed that some of the actions on Ca^2+^ influx or mobilization from cellular stores observed in the study for *Viscum album* L. might be partly mediated by NO. This is because NO inhibits Ca^2+^ influx through ligand gated Ca^2+^ channels as well as release from cellular stores. Thus, it is probable that *Viscum album* L. might achieve vasorelaxation through dual mechanisms, the NO/sGC pathway as well as through Ca^2+^-dependent mechanisms (Mojiminiyi et al. 2008) (Fig. 2). Ofem et al. (2007) suggested that the reduction in blood pressure without any alteration in heart rate by aqueous extract of leaves of *Viscum album* L. growing on citrus may be due to catecholamine-like blocking agent(s), showing predominantly alpha-1 adrenoceptor antagonist action or agonist-like agents that may be stimulating the beta-2 adrenoceptors to produce the depressor effect. In turn, Radenkovic et al. (2009) proposed that decreases in the blood pressure in rats treated with ethanolic extracts of *Viscum album* L. steams might be connected with muscarine cholinergic receptors. In rat models of myocardial infarction, flavonoids isolated from *Viscum coloratum* (Kom.) Nakai reduced ischemic myocardial injuries by blocking the signalling pathway of platelet-activating factor (PAF). A PAF antagonist isolated from mistletoe might be a homoeriodictyol-7-*O*-*β*-D-glucoside (Chu et al. 2008). Additionally, *Viscum album* L. improved haematological parameters in rats. Mistletoe extracts reduced red blood cell count and packed cell volume (Ofem et al. 2009; Ladokun et al. 2015) and brought the elevated total plasma protein levels and reduced erythrocyte sedimentation rate in the high salt-fed rats to near control levels, indicating the ability of the extract to prevent marked changes in the blood viscosity (Ofem et al. 2009).

**Table 2:** Pharmacological activity of *Viscum* species—in vivo studies

Pharmacological activity	*Viscum* species/Product	Part	Host tree	Extraction solvent	Compounds	Dose	Route of administration	Study duration	Experimental design	Results	References
Antihypertensive activity	*Viscum album* L	Fresh leaves	Citrus	Aqueous		150 mg/kg, daily	Orally	6 weeks	Normotensive, renal artery-occluded hypertensive and salt-induced hypertensive rats	Decrease in arterial blood pressure without alteration in heart rate, antihypertensive effect might involve sympathetic mechanism	Ofem et al. (2007)
	*Viscum album* L	Fresh steams		Ethanolic, ether and ethyl acetate		3.33 × 10^−5^, 1.00 × 10^−4^, 3.33 × 10^−4^, 1.00 × 10^−3^ mg/kg	Intraperitoneally		Atropine sulfate and hexocycline treated rats	Ethanolic extract exhibited activity even on the lowest dose, the ether and ethyl acetate extracts exhibited activity only by higher doses, antihypertensive effect might involve muscarinic receptors	Radenkovic et al. (2009)
	*Viscum album* L	Dried leaves	Pear *(Pyrus communis* auct. iber.*)*	Aqueous		250 mg/kg, daily	Orally by gavage	24 days	Isoproterenol-induced heart failure in rats	Improvement in all parameters of heart failure including left ventricular diameters, ejection fraction, serum NT-proBNP levels and histopathological changes; decrease in levels of NO, iNOS and hs-CRP	Karagöz et al. (2016)
	*Viscum album* L					0.6–2.8 g, daily	Orally	6 weeks	An open study in 120 patients with light to moderate hypertension (WHO grade I-II)	Decrease in systolic pressure (in rest and during physical exercise)	Committee on Herbal Medicinal Products (2012)
	*Viscum album* L					5 drops of drug every 5 min up to 4 administrations	Sublingually		264 patients with diagnosis of hypertension	Time of arterial blood pressure reduction was less for the group of patients who received the natural treatment	Montero et al. (2016)
	*Viscum album* L. mother tincture			Ethanolic extract manufactured according to Homoeopathic Pharmacopoeia of India		10 drops of drug in 30 ml of distilled water, three times a day	Orally	12 weeks	37 newly diagnosed hypertensive patients	Decrease in systolic and diastolic pressure, decrease in serum triglyceride	Poruthukaren et al. (2014)
	*Viscum articulatum* Burm. f	Dried herb	*Cordia macleodii* Hook.f. & Thomson	Methanolic		200 and 400 mg/kg, daily	Orally	4 weeks	L-NAME-induced hypertensive rats	Antihypertensive effect might be attributed to diuretic, nephroprotective and hypolipidemic actions, and might be due to the presence of triterpenoids	Bachhav et al. (2012)
	*Viscum articulatum* Burm. f	Cuticular wax			Oleanolic acid	60 mg/kg, daily	Intraperitoneally	15 days	Glucocorticoid (dexamethasone)-induced hypertensive rats	Decrease of the systolic blood pressure, which might be connected with its antioxidant and NO releasing action	Bachhav et al. (2011)
	*Viscum articulatum* Burm. f	Cuticular wax			Oleanolic acid	60 mg/kg, daily		4 weeks	L-NAME-induced hypertensive rats	Oleanolic acid did not affected NO level and its antihypertensive effect might be due to diuresis and nephroprotection	Bachhav et al. (2015)
Hematological parameters	*Viscum album* L	Fresh leaves	Citrus	Aqueous		150 mg/kg, daily	Orally	6 weeks	High salt-fed rats	Decrease in the red blood cells, packed cell volume, haemoglobin, total plasma protein levels and increase in erythrocyte sedimentation rate	Ofem et al. (2009)
	*Viscum album* L	Dried leaves	Coffee (*Coffee arabica*), kola (*Kola nitida*) and cocoa (*Theobromae cacao*)	Aqueous		400, 800, 1600 and 3200 mg/kg, daily	Orally	14 days	Healthy rats	Mistletoe parasitizing on kola significantly, in dose dependent manner, decreased platelets count, mistletoe parasitizing cocoa and coffee reduced haemoglobin concentration, all the extracts reduced packed cell volume, red blood cell and increased white blood cells	Ladokun et al. (2015)
Antiglycemic, antilipidemic and insulinotropic effect	*Viscum album* L	Dried leaves		Ethanolic		250, 500, 750, 1000 mg/kg		10 h	Normalglycemic and streptozotocin-induced diabetic rats	Reduction in fasting blood glucose level	Nwaegerue et al. (2007)
	*Viscum album* L	Dried herb	Apricot (*Armeniaca vulgaris* Lam.), pine (*Pinus nigra* J.F.Arnold), fir (*Abies bornmlleriana* Mattf.)	Aqueous and ethanolic		500 mg/kg	Orally by gastric gavage	8 days	Streptozotocin-induced diabetic rats	Antidiabetic effect of mistletoe depends on the host tree	Orhan et al. (2005)
	*Viscum album* L	Fresh leaves		Aqueous		100 mg/kg	Intravenously	3 h	Normalglycemic and streptozotocin-induced diabetic rats	No effect on glucose level in normal rats but decrease of the blood glucose level in the diabetic rats, increase of the insulin secretion in normal rats and in the diabetic group	Eno et al. (2008)
	*Viscum album* L	Dried herb	*Kola acuminate*	Methanolic		100 mg/kg, daily		3 weeks	Streptozotocin-induced diabetic rats	Reduction in fasting blood glucose level, HbA1c, serum triglyceride, urea, lactate dehydrogenase, α-amylase and low density lipoprotein cholesterol, increase of high density lipoprotein cholesterol	Adaramoye et al. (2012)
	*Viscum album* L	Dried leaves		Ethanolic		100 mg/kg, daily	Orally by gavage	10 days	Streptozotocin-induced diabetic rats	No significant difference in glucose level, reduction in oxidative stress	Turkkan et al. (2016)
	*Viscum album* L	Dried leaves	Citrus	Aqueous		150 mg/kg, daily	Orally with syringe and orogastric tube	3 weeks	Streptozotocin-induced diabetic rats	Reduction in fasting blood glucose level	Nna et al. (2013)
	*Viscum album* L	Dried leaves	Oil been (*Pentaclethra macrophylla* Benth.)	Aqueous		200 mg/kg	Intraperitoneally	4 h	Fasted normalglycemic rats	Decrease in blood glucose level	Ohiri et al. (2003)
						400 mg/kg			Alloxan-induced diabetic rabbits		
	*Viscum album* L	Dried leaves	Oak	Aqueous		500 and 1000 mg/kg, daily	Orally by gavage	3 days	Alloxan-induced diabetic rats	Decrease in serum glucose concentration and increase in the serum insulin level	Shahaboddin et al. (2011)
	*Viscum album* L	Dried leaves	Sweet orange (*Citrus sinensis* (L.) Osbeck), african pear *(Dacroydes edulis*), guava (*Psidium guajava* L.) and pepper fruit (*Dennettia tripetala* Baker f.)	Aqueous		100 mg/kg, daily	Orally by gavage	14 days	Alloxan-induced diabetic rats	The strongest activity was exhibited by extracts of mistletoe growing on *Citrus sinensis* and *Pisium guajava*	Umoh et al. (2011)
	*Viscum album* L	Dried leaves		Ethanolic		2 mg/kg, 16 h	Intraperitoneally	54 h	Alloxan-induced diabetic rats	Decrease in fasting blood glucose level	Ibegbulem and Chikezie 2013)
	*Viscum coloratum* (Kom.) Nakai	Dried herb	Oak (*Quercus variabilis* Blume)	Protein fraction		50—400 µg/ml	Intraperitoneally	10 days	Alloxan-induced diabetic mice	Decrease in the blood glucose level and volume of drinking water	Kim et al. (2014b)
	*Viscum coloratum* (Kom.) Nakai	Dried herb	Oak	Aqueous and ethanolic	Betulin and oleanolic acid	Diet containing 0.2 or 0.6% of extract	Orally	8 weeks	Partial pancreatectomized rats	Ethanolic extract made β-cell mass greater by increasing β-cell proliferation and decreasing its apoptosis	Ko et al. (2016)
	*Viscum schimperi* Engl	Dried herb		Methanolic		500 mg/kg, daily	Orally by gavage	4 weeks	Streptozotocin-induced diabetic rats	Reduction in the fasting blood glucose level; increase of the level of insulin, reduction of total cholesterol, trigliceryde and low density lipoprotein cholesterol and increase of high density lipoprotein cholesterol	Abdel-Sattar et al. (2011)
Hepatoprotective activity	*Viscum album* L	Leaves		Ethanolic		1 g/kg	Orally		Paracetamol-induced hepatotoxity in rats	Reduction of ALT, ALP levels, no influence on the levels of total bilirubin and total protein	Ogbonnanya et al. (2010)
	*Viscum album* L	Dried leaves	Cocoa (*Theobroma cacao* L.) and cola (*Cola nitida* (Vent.) Schott & Endl.)	Methanolic		1000–5000 mg/kg, daily	Orogastrically	7 days	Paracetamol-induced hepatotoxity in rats	No significant difference in AST, ALT and ALP for *V. album* growing on cocoa, significant increase in AST, ALT and ALP for *V. album* growing on cola at 4000 and 5000 mg/kg doses	Yusuf et al. (2015)
	*Viscum album* L	Dried leaves	Citrus	Aqueous		150 mg/kg, daily	Orally (syringe and or gastric tube)	6 weeks	High salt diet rats	Decrease in serum total bilirubin, serum conjugated bilirubin and serum unconjugated bilirubin	Ofem et al. (2014)
	*Viscum album* L	Dried leaves	Citrus	Aqueous		150 mg/kg, daily	Orally (syringe and or ogastric tube)	3 weeks	Streptozotocin-induced diabetic rats	Decrease in serum total bilirubin, serum conjugated bilirubin and serum unconjugated bilirubin	Nna et al. (2014)
	Viscum Fraxini-2 (*Viscum album* L.)		Ash	Aqueous		0.1 and 0.2 ml/kg, once weekly	Subcutaneously	30 days	Carbon tetrachloride-induced hepatotoxity in rats	Decrease in ALT, AST and ALP levels, restoration of the normal architecture of the liver tissue with minimal fibrosis	Abdel-Salam et al. (2010)
						0.2 ml/kg of mistletoe + 25 mg/kg of sylimarin, once weekly					
	Iscador Qu (*Viscum album* L.)	Fresh herb	Oak (*Quercus robur* L. and *Quercus petraea* (Matt.) Liebl.)	Fermented, aqueous extract	380 ng/ml of lectins, 14 mg/ml of viscotoxines	Two 5 mg ampules, three times weekly	Subcutaneously	12 months	5 patients with chronic hepatitis C	6–20 fold reduction in viral load (HCV-RNA) and complete remission of elevated AST and ALT in two out of five patients, an increase of HCV RNA in one patient	Tusenius et al. (2001)
	Iscador Qu (*Viscum album* L*.*)	Fresh herb	Oak (*Quercus robur* L. and *Quercus petraea* (Matt.) Liebl.)	Aqueous	750 ng of lectins	10 mg, three times weekly	Subcutaneously	12 months	21 patients with chronic hepatitis C	Decrease in ALT and AST during the 12 months treatment and slight increase after treatment end	Tusenius et al. (2005)
	Abnoba*Viscum* (*Viscum album* L.)	Fresh herb	Oak	Aqueous	1000 ng of lectins	0.15 mg, three times weekly					
	Abnoba*Viscum* Quercus (*Viscum album* L.)	Fresh herb	Oak	Aqueous	65–3610 ng of lectins (mean weekly dose)	Three times a week	Subcutaneously	9 months	25 patients with chronic hepatitis C and elevated alanine aminotransferase (ALT) levels	None of the patients had complete or partial normalization of ALT or HCV-RNA levels during treatment period, mean ALT did not change during the study	Huber et al. (2001)
	*Viscum coloratum* (Kom.) Nakai	Dried steams and leaves		Aqueous	Alkaloid fraction	120 mg/kg, daily	Orally by gastric gavage	8 weeks	Carbon tetrachloride-induced hepatic fibrosis in rats	Decrease of hepatic fibrosis; reduction in mRNA levels of TGF-β1, procollagen I and TIMPs; increase in TGF-β1, TGF-β1 receptor, phosphorylated Smad 2 and α-SMA proteins in liver tissues; increase in Smad 7 level	Jiang et al. (2014)
Antiepileptic activity	*Viscum album* L	Fresh leaves	Citrus	Aqueous		50 and 150 mg/kg	Orally		Maximum electro shock, isoniazid- and pentylenetetrazole-induced seizures in mice and rats	Reduction in various phases of epileptic seizures, increased latency to the first convulsion, increased convulsion onset and reduction in seizure duration	Gupta et al. (2012)
	*Viscum album* L	Dried herb	Maple (*Acer platanoides* L.)	Aqueous and aqueous-ethanolic		100 mg/kg	Intragastrically	2 days	Pentylenetetrazole-induced seizures in mice	Effective against pentylenetetrazole-induced seizures	Tsyvunin et al. (2016)
			Willow (*Salix alba* L.)	Ethanolic							
	Viscum Mali e planta tota *(Viscum album* L.*)*		Apple tree			Initially given in strength D5, 10 granules BID, equivalent to a 1:100,000 dilution of the whole plant extract, later increased to D2, equivalent to a 1:100 dilution, 10 granules twice a day		12 weeks	4½-year-old girl suffering from childhood absence epilepsy	The dose increase of Viscum Mali, in addition to an existing combination with valproic acid and clobazam, may have played a key role in achieving seizure freedom for this child	von Schoen-Angerer et al. (2015)
	*Viscum capense* L. f	Dried stems		Methanolic		50 and 100 mg/kg	Intraperitoneally		Pentylenetetrazole-, bicuculline- and N-methyl-DL-aspartic acid- induced seizures in mice	Delayed the onset of pentylenetetrazole—and bicuculline-induced seizures and reduction in the number of convulsing animals; moderate effect against N-methyl-DL-aspartic acid-induced tonic seizures	Amabeoku et al. (1998)
	*Viscum articulatum* Burm. f	Dried herb		Methanolic		100 and 200 mg/kg, daily	Orally	7 days	Maximum electro shock- and pentylenetetrazole- induced seizures in rats	Reduction in duration of hind limb extensor phase and increase in the latency to convulsions	Geetha et al. (2010)
	*Viscum articulatum* Burm. f	Fresh herb		Chloroform and methanolic	Syringaresinol	150 and 300 mg/kg for extracts, 10 and 20 mg/kg for isolated compound	Orally	7 days	Picrotoxin- induced seizures in rats	Extracts and syringaresinol delayed the onset of tonic convulsions, increase in the brain GABA levels in rats treated with the methanolic extract	Geetha et al. (2018)
									N-methyl-d-aspartic acid—induced seizures in mice	Only the methanolic extract and syringaresinol antagonized the N-methyl-d-aspartic acid -induced turning behaviour	
Sedative activity	*Viscum album* L	Fresh leaves	Citrus	Aqueous		50 and 150 mg/kg	Orally		Mice placed in actophotometer	Reduction in locomotor activity	Gupta et al. (2012)
	*Viscum album* L	Fresh leaves	Citrus	Aqueous		50 and 150 mg/kg	Orally		Pentobarbital- induced sleeping time in mice	Increase in duration of sleeping time	Gupta et al. (2012)
	*Viscum album* L	Dried herb		Methanolic and its ethyl acetate and 1-butanol fractions		200 and 400 mg/kg for extract, 25 and 50 mg/kg for fractions	Orally		Open field test on mice	Reduction in rearing and crossings	Kumar et al. (2016)
	*Viscum orientale* Willd	Dried leaves	*Exoecaria agalloch*	Methanolic		300 and 500 mg/kg	Orally		Open field test and hole cross test in mice	Reduction in spontaneous motor activities	Khatun et al. (2016)
Hypnotic activity	*Viscum album* L	Dried herb		Methanolic and its ethyl acetate and 1-butanol fractions		200 and 400 mg/kg for extract, 25 and 50 mg/kg for fractions	Orally		Thiopentone sodium induced-sleeping time assay in mice	Increase in the duration of sleep in mice	Kumar et al. (2016)
Antipsychotic activity	*Viscum album* L	Fresh leaves	Citrus	Aqueous		50 and 150 mg/kg	Orally		Apomorphine-induced stereotypy in mice and rats	Significantly reduction in the stereotyped behaviour	Gupta et al. (2012)
	*Viscum album* L	Fresh leaves	Citrus	Aqueous		50 and 150 mg/kg	Orally		Haloperidol-induced catalepsy in mice and rats (bar test)	Enhancement in cataleptic effect of haloperidol	Gupta et al. (2012)
Antianxiety activity	*Viscum album* L	Dried herb		Methanolic and its ethyl acetate and 1-butanol fractions		50 and 100 mg/kg for extract, 5 and 10 mg/kg for fractions	Orally		Elevated plus-maze test on mice (EPM model)	The number of entries and time spent in open arms in the elevated plus-maze test were significantly increased	Kumar et al. (2016)
Antistress activity	*Viscum album* L	Dried herb		Methanolic and its ethyl acetate and 1-butanol fractions		200 and 400 mg/kg for extract, 25 and 50 mg/kg for fractions	Orally		Cold swim test on mice	Reduction in time spent by mice in the immobile state	Kumar et al. (2016)
Antidepressant activity	*Viscum album* L	Dried herb		Methanolic and its ethyl acetate and 1-butanol fractions		200 and 400 mg/kg for extract, 25 and 50 mg/kg for fractions	Orally		Despair swim test on mice	Reduction in the duration of immobility in mice	Kumar et al. (2016)
Analgesic activity	*Viscum album* L	Dried herb		Methanolic and its ethyl acetate and 1-butanol fractions		200 and 400 mg/kg for extract, 25 and 50 mg/kg for fractions	Orally		Tail immersion test was conducted by recording tail withdrawal from heat (flicking response) in mice	Significant analgesic activity	Kumar et al. (2016)
	*Viscum album* L	Dried leaves and stems	Apricot (*Armeniaca vulgaris* Lam.)	Ethyl acetate	2′-Hydroxy-4′,6′-dimethoxy-chalcone-4-*O*-*β-d*-glucopyranoside and 5,7-dimethoxy-flavanone-4′-*O*-[*β*-D-apiofuranosyl-(1 → 2)]-*β*-D-glucopyranoside	125 and 250 mg/kg for extract and 30 mg/kg for isolated compounds	Orally		p-Benzoquinone-induced writhing test in mice and carrageenan-induced hind paw edema model in mice	Ethyl acetate fraction and isolated compounds exhibited antinociceptive and anti-inflammatory activity	Orhan et al. (2006)
	*Viscum orientale* Willd	Dried leaves	*Exoecaria agalloch*	Methanolic		300 and 500 mg/kg	Orally		Acetic acid-induced writhing model in mice and formalin-induced paw licking in mice	Writhing and paw licking inhibition	Khatun et al. (2016)
Alzheimer’s disease	*Viscum album* L	Dried leaves	Orange tree	Aqueous		100 mg/kg, daily	Orally	21 days	Aluminum chloride-induced Alzheimer’s disease in mice	Increase in the brain-derived neurotrophic factor (BDNF); reduction of aluminum chloride-induced memory impairment and oxidative damage	Ademola et al. (2016) and Ekpenyong et al. (2016)
	*Viscum coloratum* (Kom.) Nacai	Dried herb		Methanolic		25 and 50 mg/kg, daily	Orally	7 days	Intracerebroventricular injection of amyloid β protein in mice	Protection from memory impairment induced by intracerebroventricular injection of amyloid β protein	Jang et al. (2015)
Mood	Eurixor *(Viscum album* L.*)*	Fresh herb		Aqueous	Lectin (ML-1)	1 ng/kg body weight, twice a week	Subcutaneously	12 weeks	Breast cancer patients (n = 36)	Increased levels of plasma beta-endorphin	Heiny and Beuth 1994)
	Eurixor *(Viscum album* L.*)*	Fresh herb		Aqueous	Lectin (ML-1)	0.5–1.0 ng /kg body weight, twice a week	Subcutaneously	24 weeks	Breast cancer patients (n = 47)	Increased levels of plasma beta-endorphin	Heiny et al. (1998)
Antiobesity activity	*Viscum coloratum* (Kom.) Nacai	Dried herb	Oak	Aqueous		3 g/kg, daily	Orally	15 weeks	High-fat diet-induced obesity in mice	Reduction in body and epididymal fat pad weights	Jung et al. (2013)
	*Viscum coloratum* (Kom.) Nacai	Dried herb	Oak	Aqueous and ethanolic	Betulin and oleanolic acid	Diet containing 0.2 or 0.6% of extract	Orally	8 weeks	Partial pancreatectomized rats	Reduction in epididymal fat mass by increasing fat oxidation	Ko et al. (2016)
Endurance capacity	*Viscum coloratum* (Kom.) Nacai	Dried herb	Oak	Aqueous		3 g/kg, daily	Orally	15 weeks	Endurance test with treadmill in high-fat diet- induced obesity mice	Mistletoe treated mice run twice as far as high-fat diet mice	Jung et al. (2013)
	*Viscum coloratum* (Kom.) Nacai	Dried herb	Oak	Aqueous		400 and 1000 mg/kg, daily		1 week	Endurance test with treadmill in mice	Mistletoe treated mice run 2.5-times longer than control mice, plasma lactate levels of exhausted mice were significantly lower	Jung et al. (2012)
						25—400 mg/kg, daily			Forced swim test in mice	The swimming time to exhaustion was prolonged by as much as 212%	
	*Viscum coloratum* (Kom.) Nacai	Leaves	Oak	Aqueous		500 mg/kg, daily	Orally	2 weeks	Endurance test with treadmill in mice	Decreases in level of plasma lactate dehydrogenase, increase in the plasma FFA level	Lee et al. (2014)
	*Viscum coloratum* (Kom.) Nacai	Whole plant		Aqueous		diet Containing 0.3 and 1.5% of extract	Orally	4 weeks	Treadmill and swimming pool tests in mice	Increased swimming activity and elevated running times on the treadmill	Jeong et al. (2017)
Activity against muscle decline	*Viscum coloratum* (Kom.) Nacai	Whole plant		Aqueous		200 and 500 mg/kg, twice a day	Orally by gavage	15 days	Denervated mice	Decrease in denervation, decrease in the expression of Atrogin-1, no effect on Murf1 expression	Jeong et al. (2017)
						Diet containing 0.3 and 1.5% of extract		4 weeks	Mice	Increased whole body weights, a higher weight of quadricepses, increased grip strengths, increased swimming activity and elevated running times on the treadmill, increased skeletal muscle area and diameter	
	*Viscum coloratum* (Kom.) Nacai	Whole plant		Aqueous		1 and 2 g/d	Orally	12 weeks	Randomized controlled trial with 67 patients aged 55–75	Significant differences were found in atrogin-1 mRNA, myogenin mRNA and insulin growth factor 1 receptor phosphorylation	Lim et al. (2017)
Nephroprotective activity	Helixor M (*Viscum album* L.)	Fresh herb	Apple tree (*Malus domestica* Borkh.)	Aqueous		5 mg/kg	Intraperitoneally	10 days	Methotrexate-induced acute oxidative stress and nephrotoxicity in rats	Improvement in the glutathione peroxidase and superoxide dismutase activities, decrease in the NO and myeloperoxidase levels was not significant	Sakalli Çetin et al. (2017)
	*Viscum articulatum* Burm. f		*Cordia macleodii* Hook.f. & Thomson		Oleanolic acid	40, 60 and 80 mg/kg, daily	Orally	8 days	Gentamicin-induced nephrotoxicity	Decrease in serum and urine levels of creatinine, albumin and urea	Patil et al. (2010)
Diuretic activity	*Viscum angulatum* B.Heyne ex DC	Dried herb	*Randia dumetorum* (Retz.) Lam	Methanolic		100, 200 and 400 mg/kg	Orally	24 h	Rats	Dose-dependent increase in urine excretion volume, significant saluretic and natriuretic activity, the Cl(-)/Na( +) + K( +) ratio, which indicates carbonic anhydrase mediated activity remained unaffected	Jadhav et al. (2010b)
	*Viscum articulatum* Burm. f	Dried herb	*Cordia macleodii* Hook.f. & Thomson	Methanolic		100, 200 and 400 mg/kg	Orally	24 h	Rats	Dose-dependent increase in urine excretion volume, significant saluretic and natriuretic activity	(Jadhav et al. (2010a)
Wound healing	*Viscum album* L			Liphohilic extract		Ointment	Topical treatment		12 patients with 15 BCC lesions	Achievement of hemostasis in bleeding tumor wounds and after a prolonged treatment period a wound epithelialization with a thin epithelial layer	Kunz et al. (2011) and Kuonen et al. (2013)
	*Viscum articulatum* Burm. f	Whole plant		Ethanol		1% extract ointment			Incision, excision and dead space wound model in rats	Reduction in wound area, faster re-epithelization rate	Garg et al. (2012)
Antiulcer activity	*Viscum articulatum* Burm. f	Dried herb		Methanolic		200 and 400 mg/kg	orally		Ethanol-induced ulcer model and pylorus ligation ulcer model in rats	Inhibition of the gastric lesions	Naganjaneyulu et al. (2011)
Antibacterial activity	*Viscum album* L	Dried leaves	Cocoa	Methanolic		1000 mg/kg, daily		7 days	Rats with infections of *Staphylococcus aureus* + *Bacillus cereus*, *Escherichia coli* + *Pseudomonas aeruginosa*, *S. aureus* + *P. aeruginosa* and infection of *E. coli*	Heamatological and histopathological analyses showed therapeutic effects of the extract	Yusuf et al. (2013)

## Antidiabetic activity

**Fig. 3:**
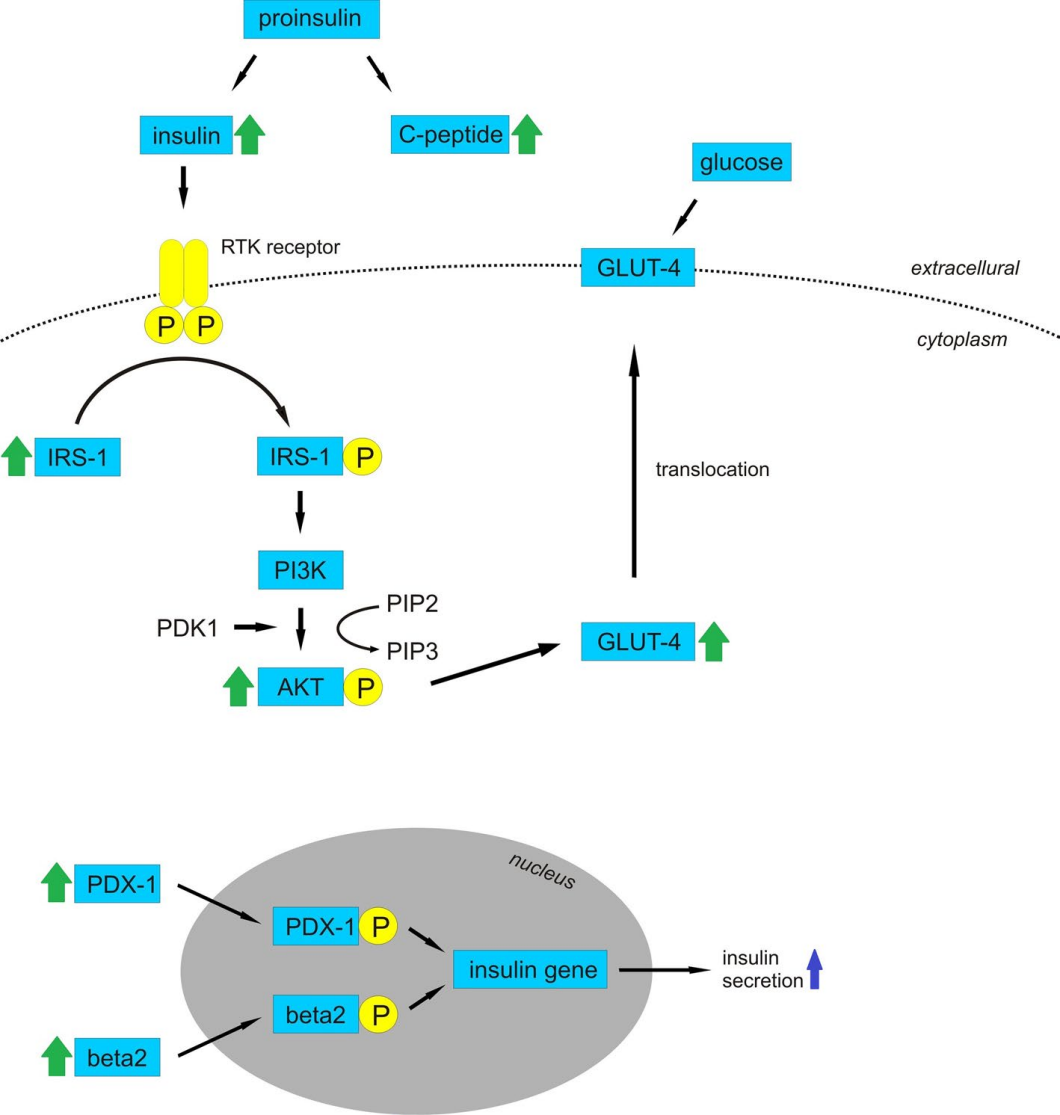
Mechanism of antidiabetic activity of mistletoe. Mistletoe increases the secretion of insulin and insulin precursor, C-peptide. Insulin binds to tyrosine kinase receptor (RTK). The activated receptor phosphorylates the IRS-1 protein leading to activation of PI3K which catalyzes the addition of phosphate group to PIP2, converting it to PIP3. PIP3 activates PDK1 leading to AKT phosphorylation, recruitment of the glucose transporter GLUT-4 to the membrane and glucose inflow. Mistletoe enhances the expression of GLUT-4, IRS-1 and AKT. Furthermore, protein fraction of mistletoe upregulates transcription factors PDX-1 and beta2 (neuroD). PDX-1 and beta2 become phosphorylated (this process might be mediated by PI3K and ERK1/2 pathways) and regulate insulin gene transcription

In vivo studies on rats showed that *Viscum* species exhibit antiglycemic and insulinotropic activity by decreasing blood glucose level and increasing the insulin secretion (Ohiri et al. 2003; Nwaegerue et al. 2007; Eno et al. 2008; Shahaboddin et al. 2011; Abdel-Sattar et al. 2011; Adaramoye et al. 2012; Ibegbulem and Chikezie 2013; Kim et al. 2014b; Turkkan et al. 2016) (Table 2). Furthermore, the effects of mistletoe have been shown to be dependent on host trees (Orhan et al. 2005; Umoh et al. 2011). The antilipidemic activity of mistletoe was shown in the reduction in low density lipoprotein cholesterol (LDL) and the increase in high density lipoprotein cholesterol (HDL) (Abdel-Sattar et al. 2011; Adaramoye et al. 2012; Kim et al. 2015) as well as improvement of HOMA-IR (Homeostatic Model Assessment of Insulin Resistance), which is an indicator of insulin resistance (Kim et al. 2015). Gray and Flatt (1999) showed that aqueous extract of *Viscum album* L. exhibited dose-dependent activity to stimulate insulin secretion by rat clonal pancreatic β-cells, and the effect was not mediated by lectins. Furthermore, Kim et al. (2014b) showed that Korean mistletoe growing on oak increased the insulin secretion from the rat pancreatic β-cells (RINm5F cells) without any effects of cytotoxicity. The lectin-free protein fraction induced insulin secretion was similar to the Korean mistletoe extract. It was also reported that the protein fraction upregulated pancreatic and duodenal homeobox 1 (PDX-1) and beta2 (neuroD), which are transcription factors regulating the expression of insulin gene. An ethanolic extract of Korean mistletoe growing on oak also made β-cell mass greater by increasing β-cell proliferation and decreasing its apoptosis. An in vitro study showed that betulin potentiated insulin-stimulated glucose uptake by increasing PPAR-γ (peroxisome proliferator-activated receptor γ) activity and insulin signalling in 3T3-L1 adipocytes, whereas oleanolic acid enhanced glucose-stimulated insulin secretion and cell proliferation in insulinoma cells (Ko et al. 2016). Aqueous *Viscum coloratum* (Kom.) Nakai extract significantly increased the secretion of insulin and an insulin precursor, C-peptide, by RINm5F cells. In differentiated C2C12 cells, the extract enhanced the expression of glucose transporter type 4 (GLUT-4), insulin receptor substrate 1 (IRS-1), and protein kinase B (AKT), which are involved in the glucose uptake signalling pathway. Viscothionin, a polypeptide isolated from mistletoe, increased the level of insulin secretion by more than 20-fold compared to that induced by the extract (Park et al. 2019). Furthermore, it was reported that mistletoe extracts inhibited α-glucosidase activity, an enzyme catalysing the cleavage of glucose from disaccharide, impeding the digestion and adsorption of glucose, eliciting attenuated postprandial plasma glucose levels (Önal et al. 2005; Park et al. 2019). The mechanism of action is shown on Fig. 3.

## Hepatoprotective activity

**Fig. 4:**
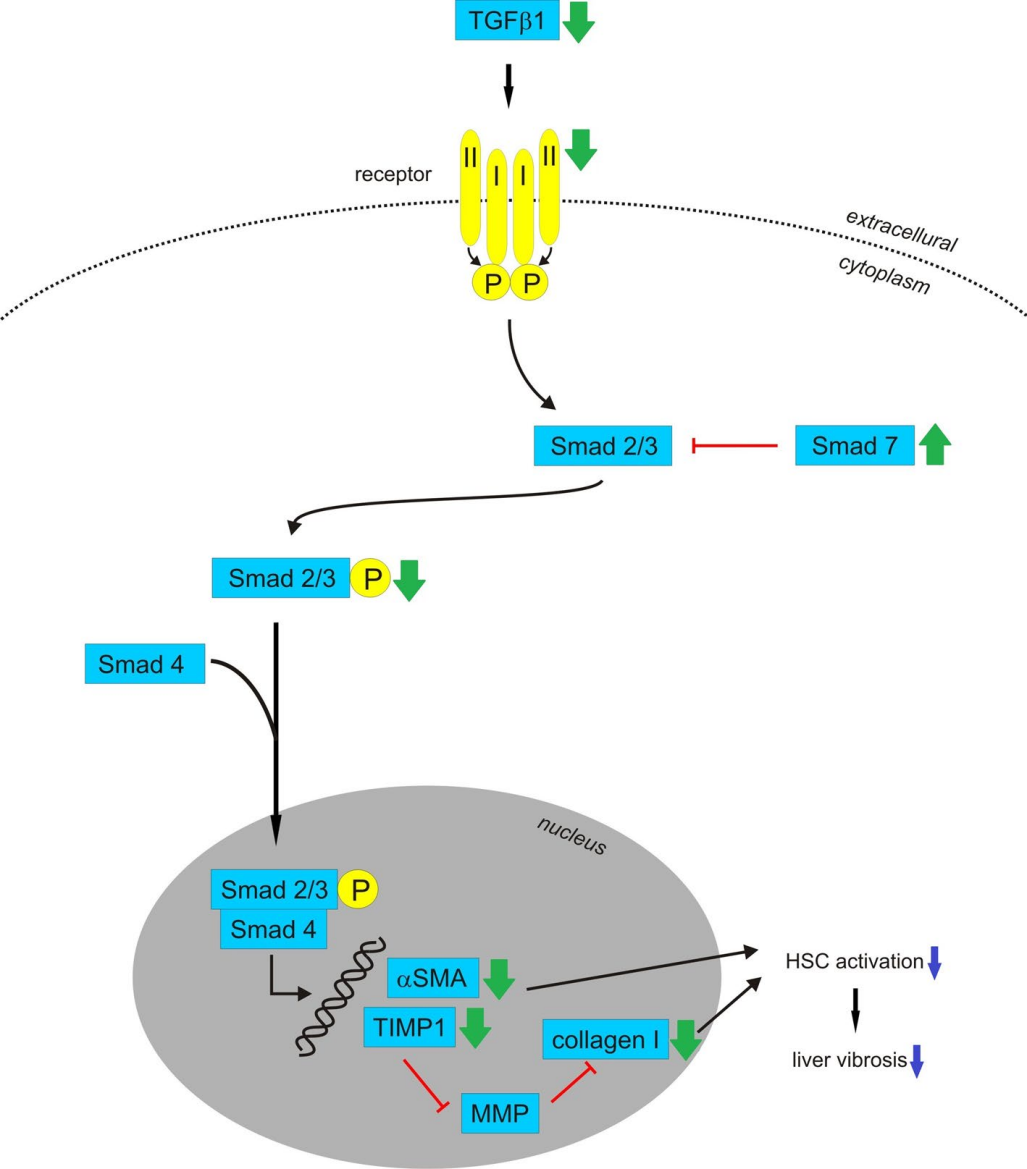
Mechanism of hepatoprotective activity of mistletoe, TGFβ/Smad pathway. TGFβ1 binds to its receptor, which consists of two type I and two type II subunits. Type II subunit phosphorylates type I subunit, which then phosphorylates Smad 2 and Smad 3. Phosphorylated Smad 2 and Smad 3 bind with Smad 4 and together they move into the nucleus to regulate expression of target genes. Smad 7 is an inhibitory Smad that negatively regulates Smad 2/3 activation. In vivo study showed that mistletoe alkaloid fractions downregulate TGF-β1, TGF-β1 receptor, phosphorylated Smad 2 and α-SMA proteins as well as downregulate the mRNA levels of TGF-β1, collagen I and TIMP-1. In contrast, Smad 7 level is upregulated. In vitro study showed that mistletoe alkaloid fractions induce Smad 7 expression and inhibit the expression of α-SMA, TGFβ1, TGF-β1 receptor, Smad 2 and TIMP-1

Indicators of liver cell injury are increased levels of serum aminotransferases, alanine aminotransferase (ALT) and aspartate aminotransferase (AST) as well as alkaline phosphatase (ALP). Studies on rats with hepatic damage showed that *Viscum* species decreased levels of ALT, AST and ALP (Abdel-Salam et al. 2010; Ogbonnanya et al. 2010; Yusuf et al. 2015) (Table 2). Furthermore, aqueous extracts of leaves of *Viscum album* L. growing on citrus decreased levels of serum bilirubin in high-salt-fed rats and in streptozotocin-induced diabetic rats (Nna et al. 2014; Ofem et al. 2014). The results of studies on patients with chronic hepatitis C were ambiguous. Treatment with either Iscador or Abnoba*Viscum* caused significant improvement for AST and ALT (Tusenius et al. 2005). Furthermore, in two out of five patients treated with Iscodar, a 6–20 fold viral load reduction (HCV-RNA) and improvements for AST and ALT were observed (Tusenius et al. 2001). On the other hand, none of the patients with chronic hepatitis C and elevated ALT levels had complete or partial normalization of ALT or HCV-RNA levels during treatment with Abnoba*Viscum* Quercus (Huber et al. 2001). The mechanism of hepatoprotection by mistletoe is not clear, but it might be mediated by the TGF-β/Smad pathway (Fig. 4). It is accepted that hepatic fibrosis is characterized by an excessive accumulation of extracellular matrix (ECM) proteins. Transforming growth factor-β1 (TGF-β1) is a cytokine leading to the activation of hepatic stellate cells (HSCs), and it stimulates ECM production while inhibiting its degradation. Once activated, TGF-β1 binds its cognate receptors and functions in autocrine and paracrine manners to exert its activities via Smad-dependent and -independent pathways. Smads are signal transduction molecules transmitting signals directly from cell surface receptors to the nucleus. Smad signal transduction pathways are thought to mediate TGF-β1-induced collagen synthesis and to play a crucial role in the process of liver damage. Nine Smads have been reported and classified into three groups. When TGF-β1 binds to its receptor, Smad 2/3 is phosphorylated and binds with Smad 4, and they move together into the nucleus for translation and expression of the target gene. Smad 7 is an inhibitory Smad that negatively regulates Smad 2/3 activation and functions by targeting the TGF-β1 receptor. An in vivo study on rats with carbon tetrachloride-induced hepatotoxicity showed that TGF-β1, TGF-β1 receptor and phosphorylated Smad 2 protein levels were reduced and Smad 7 level was increased after treatment with mistletoe alkaloid fractions. The mRNA levels of collagen I and tissue inhibitors of metalloproteinases (TIMP-1) were also downregulated. Collagen I is the prototype constituent of the fibril-formatting matrix in fibrotic liver, whereas TIMP-1 is an endogenous inhibitor of the matrix metalloproteinase (MMP) degradation of ECM. Furthermore, the mistletoe alkaloid fractions blocked α-SMA (α smooth muscle actin), the marker of activated HSC. An in vitro study on HSC-T6 cells showed that treatment with mistletoe alkaloid fractions induced Smad 7 expression and inhibited the expression of α-SMA, TGFβ1, TGF-β1 receptors, Smad 2 and TIMP-1 (Jiang et al. 2014).

## Neuropharmacological activity

**Fig. 5:**
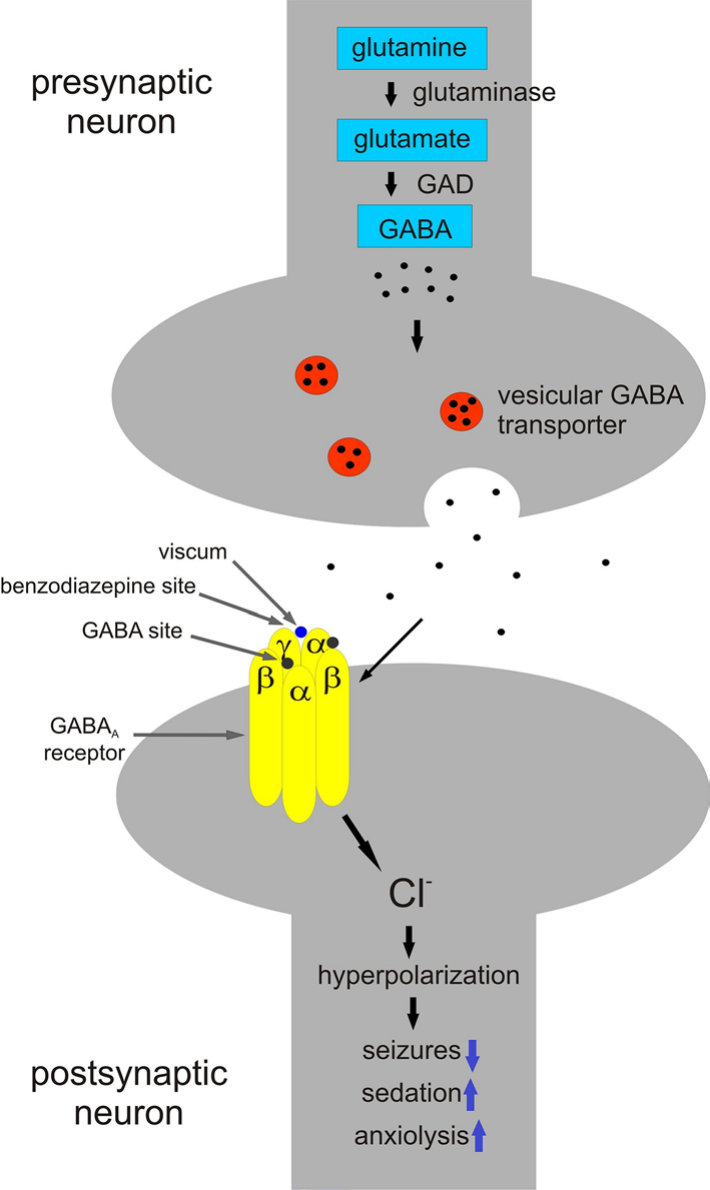
Mechanism of neuropharmacological activity of mistletoe, GABAergic signalling. Mistletoe compounds might be positive allosteric modulators of GABA_A_ receptor. They might bind to benzodiazepine site increasing the binding affinity of the receptor for GABA. This results in increased frequency of chloride ion channel opening, increased influx of chloride ions and hyperpolarization leading to anticonvulsant, sedative and anxiolytic activity

**Fig. 6:**
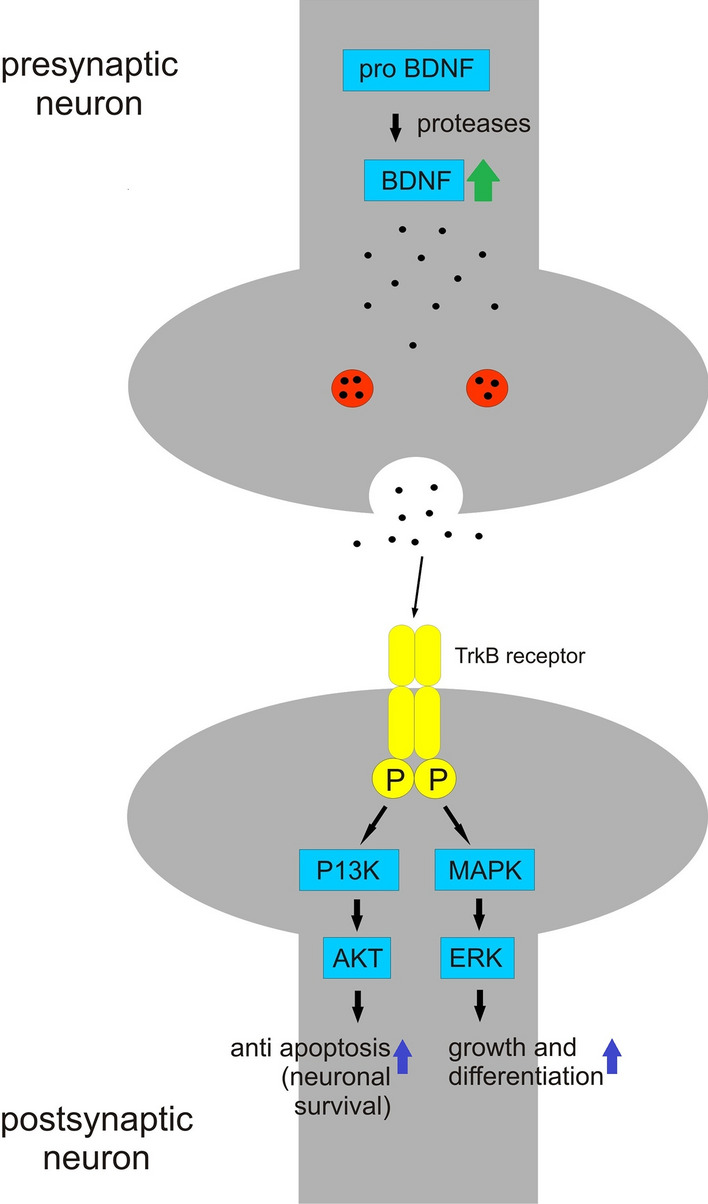
Mechanism of neuropharmacological activity of mistletoe, BDNF signalling. Mistletoe has been reported to increase brain-derived neurotrophic factor (BDNF) level. BDNF binds to tyrosine receptor kinase B leading to its phosphorylation and activation of signaling pathways. The P13K pathway activates AKT leading to neuronal survival whereas MAPK/ERK pathway leads to neuronal growth and differentiation

The influence of *Viscum* species on the central nervous system (CNS) is differential, and it was reviewed by Szurpnicka et al. (2019) In vivo studies on mice and rats showed that *Viscum* species exhibited antiepileptic (Amabeoku et al. 1998; Geetha et al. 2010, 2018; Gupta et al. 2012; Tsyvunin et al. 2016), sedative (Gupta et al. 2012; Khatun et al. 2016; Kumar et al. 2016), analgesic (Orhan et al. 2006; Khatun et al. 2016), antianxiety, antidepressant, hypnotic, anti-stress (Kumar et al. 2016) and antipsychotic activity (Gupta et al. 2012) (Table 2). Several studies have proposed that the CNS activity of mistletoe is mediated by GABA (γ-aminobutyric acid) receptors. GABA is the most important inhibitory neurotransmitter in the human central nervous system. GABA is involved in epilepsy, sedation and anxiolysis, and it works by binding to GABA_A_ receptors. GABA_A_ receptors are heteromeric GABA-gated chloride channels. The transmembrane ion channel is opened by a stimulus generated by GABA, which allows an influx of chloride ions. This results in a decrease of the depolarizing effects of an excitatory input, thereby depressing excitability. As a result, the cell is inhibited and an anticonvulsant, sedative or anxiolytic activity is achieved. The type of activity obtained depends on the subtype of the receptor. The GABA_A_ receptor consists of five subunits, made up of two α, two β and one γ or δ subunit. Several isoforms exist (α1–α6, β1-β3, γ1–γ3, δ), potentially giving a vast number of combinatorial mixes. However, only ten subunit combinations make up the physiologically relevant GABA_A_ receptors in the brain (Jäger and Saaby 2011). In addition to GABA binding sites, the GABA_A_ receptor possesses binding sites for compounds that allosterically modify the chloride channel gating of GABA, such as benzodiazepines and barbiturates. Benzodiazepine site agonists increase the GABA-induced chloride channel opening frequency and have established efficacy in the treatment of anxiety, insomnia and epilepsy as well as muscle relaxant, sedative, hypnotic, and cognition impairing effects (Diniz et al. 2015) (Fig. 5). Furthermore, it has been reported that mistletoe extract standardized for galactoside-specific lectin (ML-1) increases the beta-endorphin plasma levels in breast cancer patients (Heiny and Beuth 1994; Heiny et al. 1998). Endorphins act through opiate receptors. Three major type of opioid receptors have been identified, mu (μ), delta (δ) and kappa (κ). Beta-endorphin has a relatively high affinity at mu and delta receptors. Mu (μ) (agonist morphine) receptors are responsible for supraspinal analgesia, respiratory depression, euphoria, sedation, decreased gastrointestinal motility and physical dependence (Sharma et al. 2015). Treatment with an aqueous extract of *Viscum album* L. might also increase brain-derived neurotrophic factor (BDNF) (Ademola et al. 2016; Ekpenyong et al. 2016). BDNF plays a prominent role in modulating cognition and memory. BDNF is a neurotrophin that belongs to a family of proteins that promote the survival, functions and development of neurons. BDNF enhances neurogenesis and neurotransmission across the synapses, promotes synaptic growth and modulates synaptic plasticity (Fig. 6). BDNF also induces hippocampal long-term potentiation, which is important for memory formation. It was found that higher peripheral BDNF levels protect the older adults against Alzheimer’s disease (Ng et al. 2019).

## Antiobesity activity

**Fig. 7:**
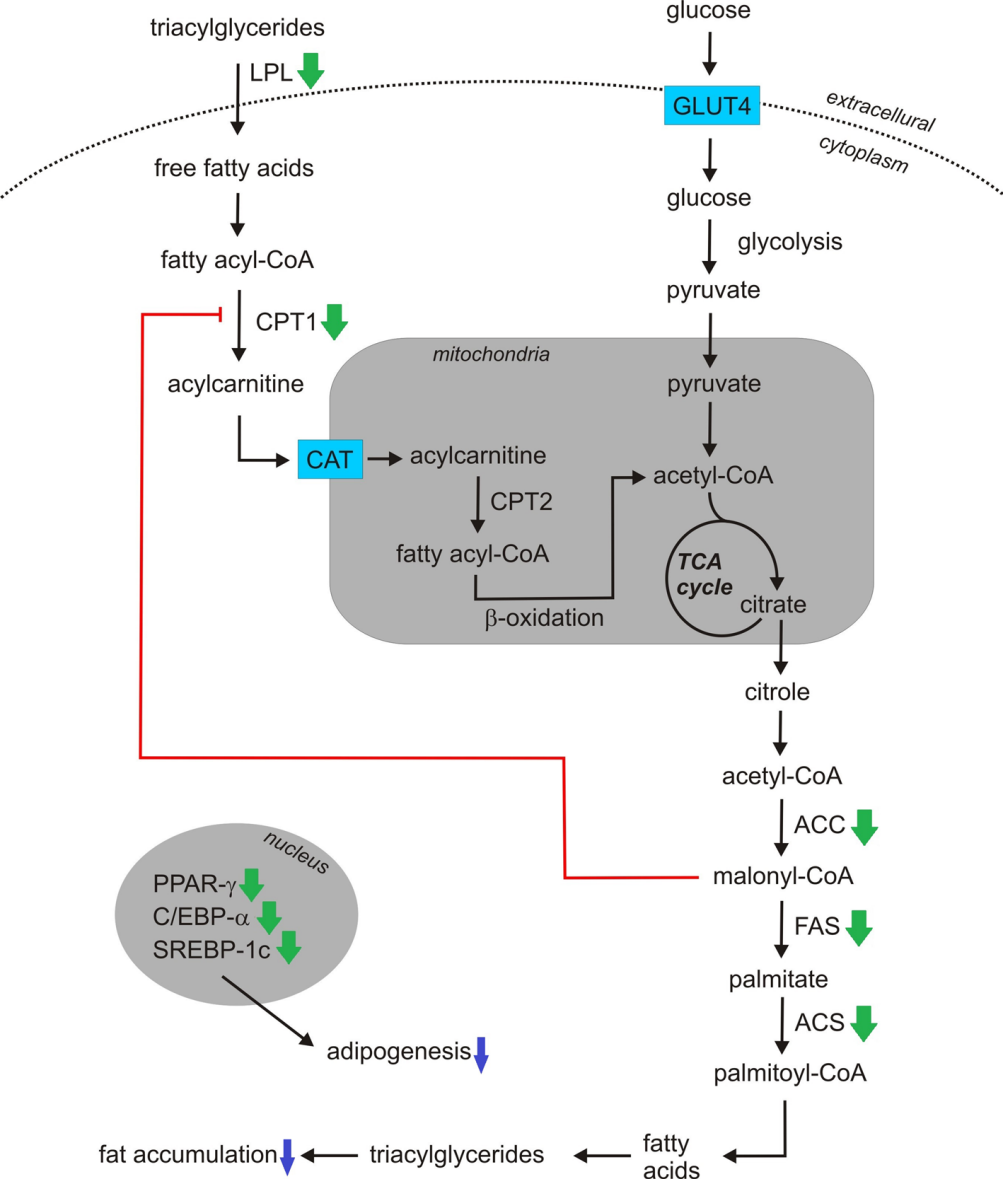
Probable mechanism of antiobesity activity of mistletoe. LPL converts triacylglycerides into free fatty acids. Free fatty acids are moved into the cell and activated to acyl-CoA. CPT1 converts acyl-CoA to acylcarnitine, which is transported into the mitochondria by CAT. CPT2 converts acylcarnitine back to acyl-CoA, and then acyl-CoA enters β-oxidation pathway. Acetyl-CoA goes into TCA cycle. Citrate exits mitochondria and is converted to acetyl-CoA, which is carboxylated to malonyl-CoA by ACC. FAS undergoes the reductive synthesis of palmitate which is converted to palmitiyl-CoA leading to formation of triacylogliceryes. Additionally, malonyl-CoA inhibits CPT-1. Mistletoe decreases expression of FAS, ACC, ACS and LPL and decreases SREBP-1c, C/EBP-α, and PPAR-γ mRNA expression

Treatment with mistletoe parasitizing oak might influence body and epididymal fat pad weights in vivo and inhibit adipogenic factors in vitro (Table 2). It is known that obesity is related with adipocyte differentiation and the extent of subsequent fat accumulation. Adipogenesis can be induced through the action of enzymes, such as fatty acid synthase (FAS), acyl-CoA synthase (ACC) and acyl-CoA synthetase (ACS). The expressions of these genes are regulated by transcription factors, including peroxisome proliferator-activated receptor γ (PPAR-γ), CCAAT/enhancer-binding protein-α (C/EBP-α) and sterol regulatory element binding element protein-1c (SREBP-1c), which are known to be crucial activators for adipogenesis and show early changes in gene expression during adipocyte differentiation (Jung et al. 2013). It has been shown that mistletoe treatment significantly decreased SREBP-1c, C/EBP-α, and PPAR-γ mRNA expression in cultured 3T3-L1 adipocytes and inhibited expression of adipocyte-specific proteins—FAS, ACC, ACS, and LPL (lipoprotein lipase) (Jung et al. 2013). Furthermore, in ovariectomized rats fed a high-fat diet, Korean mistletoe decreased FAS and SREBP-1c expression as well as increased carnitine palmitoyltransferase-1 (CPT-1) expression, a key regulator of fatty acid oxidation (Kim et al. 2015) (Fig. 7).

## Muscle mitochondrial activity

**Fig. 8:**
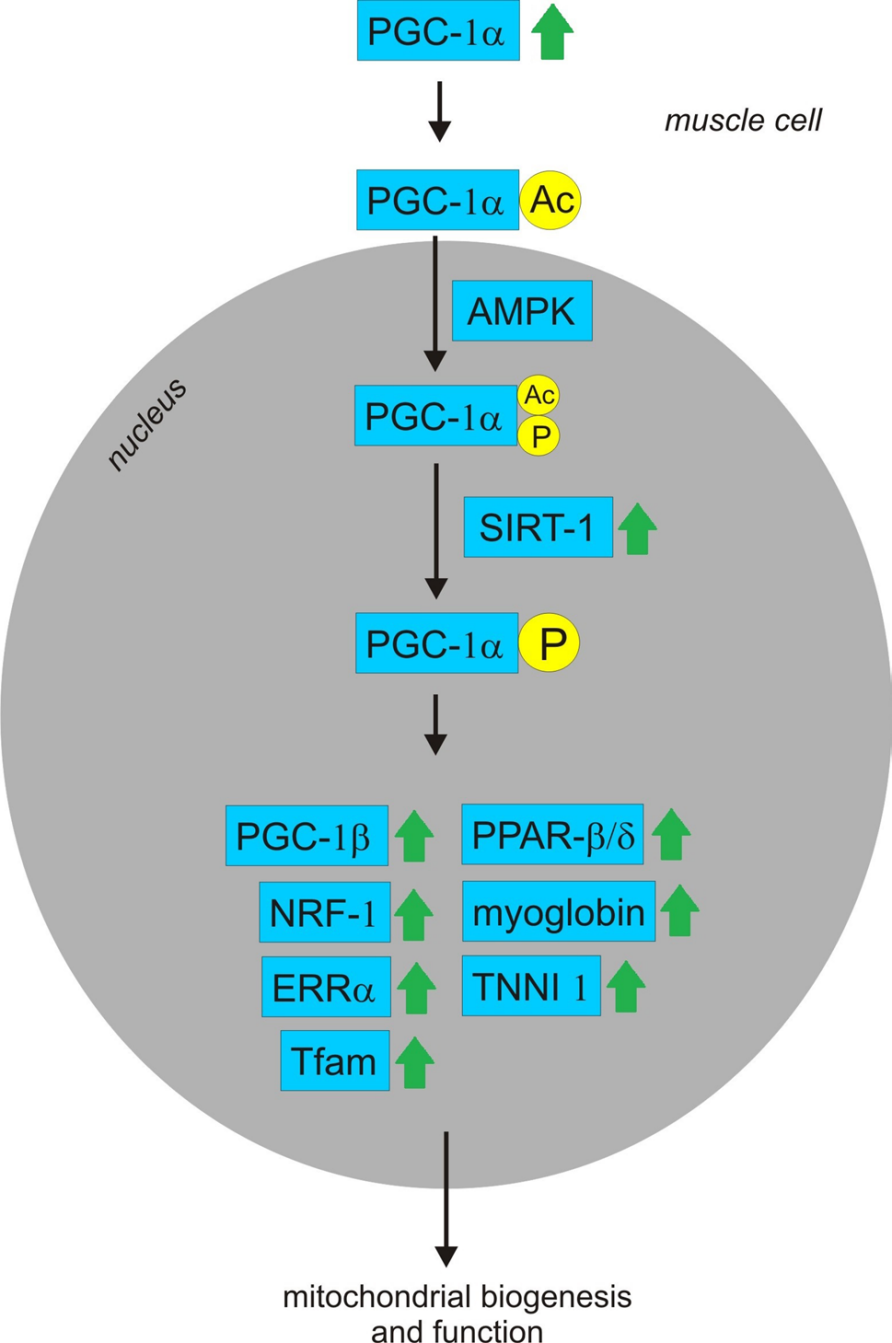
Probable effect of mistletoe on muscle mitochondrial activity. Two major genes related to mitochondrial biogenesis and function are SIRT-1 and PGC-1α. PGC-1α translocates into the nucleus where it is phosphorylated by AMPK and deacetylated by SIRT-1. Once phosphorylated and deacetylated, PGC-1α activity is increased, leading to increased transcription of mitochondrial genes. Korean mistletoe increases the mRNA expression of PGC-1α and SIRT-1 and increases the expression of PGC-1α transcriptional targets such as PGC-1β, NRF-1, ERRα, Tfam, PPARβ/δ, myoglobin and TNNI2

It was determined that the administration of an aqueous extract of Korean mistletoe might enhance exercise performance in mice (Jung et al. 2012, 2013; Lee et al. 2014) (Table 2). Jung et al. (2012) showed that an increase in endurance capacity might be mediated by improvement of mitochondrial biogenesis (Fig. 8). Korean mistletoe treatment significantly increased the mitochondrial oxygen consumption rate (OCR) in L6 cells (rat myoblast cell line) as well as increased the mRNA expression of peroxisome proliferator-activated receptor γ coactivator (PGC-1α) and silent mating type information regulation 2 homolog 1 (SIRT-1), two major genes related to mitochondrial biogenesis and function in C2C12 cells (mouse myoblast cell line). Korean mistletoe treatment increased the expression of PGC-1α transcriptional targets, such as PGC-1β, NRF-1 (nuclear respiratory factor-1), ERRα (estrogen-related receptor α), Tfam (mitochondrial transcription factor A), PPARβ/δ (peroxisome proliferator–activated receptor β/δ), MB (myoglobin) and TNNI2 (troponin I) in C2C12 cells. Additionally, Korean mistletoe decreased levels of plasma lactate and lactate dehydrogenase, parameters of tissue damage and muscle fatigue in exhausted mice (Jung et al. 2012; Lee et al. 2014). Furthermore, exercise training increases the muscular glycogen and plasma free fatty acid (FFA) level, and Korean mistletoe administration increased the plasma FFA level, indicating that Korean mistletoe administration alters the energy resources in muscle (Lee et al. 2014).

## Activity against muscle decline

**Fig. 9:**
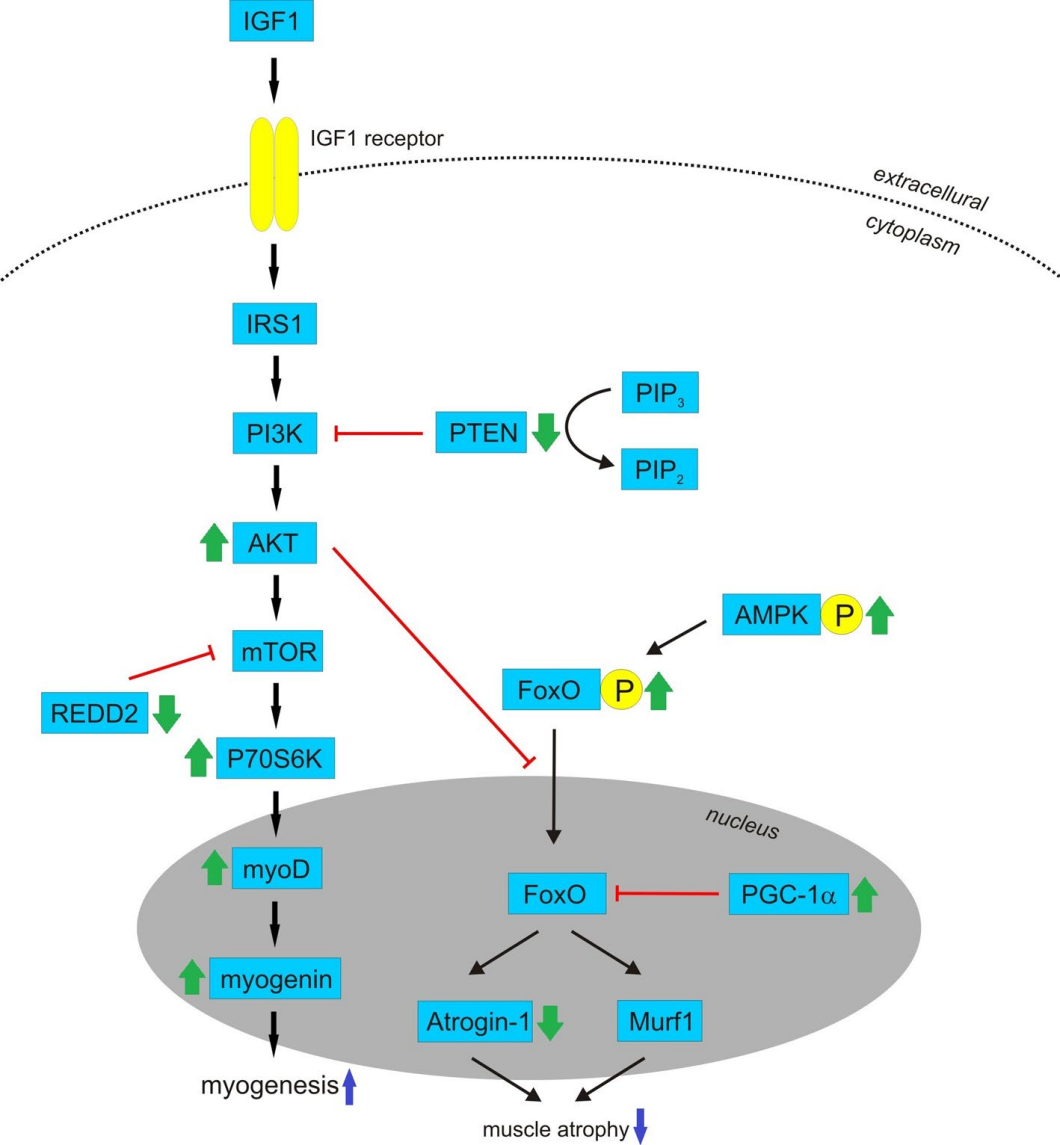
Probable effect of mistletoe against muscle decline. IGF1 binds to IGF1 receptor leading to activation of PI3K/AKT/mTOR pathway. Mistletoe leads to higher phosphotylation of AKT resulting in activation of P70S6K, upregulation of myoD and myogenin expression and myogenesis. Mistletoe decreases phosphorylation of PTEN which dephosphorylates PIP_3_, increasing PIP_2_ level and resulting in a decreased AKT activity. Furthermore, mistletoe decreases expression of REDD2 which inhibits mTOR pathway*.* Mistletoe increases phosphorylation of FoxO supporting the observation that mistletoe could induce the phosphorylation of AMPK, which is a repressor of FoxO. AKT also causes phosphorylation and nuclear exclusion of FoxO which is key molecule inducing muscle atrophy by stimulating Murf1 and Atrogin-1. Additionally, FoxO-dependent activation of muscle atrophy is inhibited by PGC-1α

Supplementation with mistletoe might be effective against age-related decline in muscle mass (Table 2). An in vitro study showed that an aqueous extract of Korean mistletoe caused higher phosphorylation of AKT in C2C12 cells (mouse myoblast cell line), suggesting that mistletoe has an effect on the regulation of the muscle mass through the activation of the AKT/mTOR (protein kinase B/ mammalian target of rapamycin) signalling pathway (Jeong et al. 2017) (Fig. 9). Furthermore, mistletoe showed increased phosphorylation of FoxO (forkhead box transcription factors of the class O) supporting the observation that mistletoe could induce the phosphorylation of AMPK (AMP-activated protein kinase), which is a repressor of FoxO. FoxO is a key molecule inducing muscle atrophy by stimulating the E3 ubiquitin ligases Murf1 and Atrogin-1. In C2C12 cells, as well as in denervated mice, mistletoe decreased gene expression of Atrogin-1. On the contrary, in C2C12 cells, mistletoe increased mRNA expression of PGC-1α, GLUT-4, and SREBP-1c genes related to the inhibition of muscle atrophy and related to the induction of muscle hypertrophy by regulating the expression of Atrogin-1and Murf1 (Jeong et al. 2017). Lim et al. (2017) conducted randomized controlled trial confirming that supplementation with Korean mistletoe extract and exercise affects muscle mass and functional capabilities. Supplementation with tablets containing aqueous extracts of *Viscum coloratum* (Kom.) Nakai was effective for suppressing intracellular pathways related to muscle protein degradation, but stimulated those related to myogenesis. The mRNA expressions levels related to muscle protein degradation (REDD2, TSC2, FoxO1, and atrogin-1) and myogenesis (mTOR, S6K1Rheb, c-Myc, myogenin, and MyoD) as well as the phosphorylation of proteins related to muscle protein degradation (GSK3β, GSK3α, TSC2, and PTEN) and myogenesis (IGF1R, IR, IRS-1, AKT, mTOR, P70S6K, RPS6 and ERK) were studied. Significant differences were found in atrogin-1 mRNA, myogenin mRNA and insulin growth factor 1 receptor phosphorylation. A single administration of mistletoe induced decreases in atrogin-1 gene expression and PTEN (phosphatase and tension homolog) phosphorylation and an increase in myogenin gene expression. A 12-week treatment induced consistent changes in atrogin-1 and myogenin gene expression. Furthermore, the increase of REDD2 gene expression and a decrease of IGF1R phosphorylation shown by the placebo group were retarded in the mistletoe treated group at a 12-week administration. In patients treated with mistletoe, along with an endurance exercise program, the body composition was significantly changed, and knee strength and the dynamic balance ability were improved (Lim et al. 2017).

## Antioxidative activity

Oxidative stress, defined as an imbalance between oxidants and antioxidants in favour of oxidants, leads to many biochemical changes in organisms and is an important contributing factor in several human chronic diseases, such as atherosclerosis and cardiovascular diseases, mutagenesis and cancer, several neurodegenerative disorders, and aging process (Frei 1999). It is suggested that increasing intake of dietary antioxidant may help to maintain a tolerable antioxidant status and help in the disease prevention (Nimse and Pal 2015). Numerous mistletoe extracts and isolated lectin showed radical-scaveging activity and protective effects against oxidative stress induced by free radicals, nitric oxide and superoxide anion (O_2_^−^) (Sengul et al. 2009; Papuc et al. 2010; Kim et al. 2010, 2016; Kusi et al. 2015). It was studied that the activity of the more polar extracts was higher in different antioxidant mechanism and might result from high level of phenolic and flavonoid compounds (Orhan et al. 2014; Khatun et al. 2016; Pietrzak et al. 2017). Furthermore, antioxidant activity of *Viscum* species depends on host tree (Vicas and Prokisch, 2009; Vicas et al. 2011; Orhan et al. 2014; Pietrzak et al. 2017) and the time of harvest (Önay­Uçar et al. 2006).

## Nephroprotective and antidiuretic activity

Helixor M, extract from *Viscum album* L. growing on apple tree, showed activity against methotrexate (MTX)-induced acute oxidative stress and nephrotoxicity in rats. The mechanism included antioxidant and anti-inflammatory properties, as evident from significant increase in the activities of the antioxidative enzymes, superoxide dismutase (SOD) and glutathione peroxidise (GSH-Px) (Sakalli Çetin et al. 2017). Oleanolic acid isolated from *Viscum articulatum* Burm. f. showed protective effects on gentamicin-induced renal damage in rats. Oleanolic acid decreased creatinine, albumin and urea levels in the serum and urine. It protected the rat kidneys from histological alterations induced by gentamicin and improved the glomerular filtration rate. The mechanism might be due to antioxidant and diuretic activity (Patil et al. 2010). Diuretic activity was tested for *Viscum angulatum* B. Heyne ex DC. and *Viscum articulatum* Burm. f. It was showed that mistletoe had a significant effect on the urine excretion volume. The higher natriuretic activity (Na^+^/K^+^) observed suggested a potassium-sparing diuretic effect. Furthermore, the extract showed less influence on the ion quotient (Cl^−^/Na^+^ + K^+^) which suggested no inhibition of carbonic anhydrase (Jadhav et al. 2010a, b).

## Wound healing

*Viscum articulatum* Burm. f., extract showed reduction in wound area in an excision wound model in rats (Table 2). Furthermore, the re-epithelization rate was found to be faster and granuloma breaking strength as well as dry granulation tissue were significantly increased in extract-treated rats (Garg et al. 2012). Kunz et al. (2011) showed in a prospective case series study, wound healing promoting and anti-tumour effects by the topical treatment of basal cell carcinoma with ointment containing *Viscum album* L. lipophilic extract. More specifically, an achievement of haemostasis in bleeding tumour wounds, and after a prolonged treatment period, a wound epithelialization with a thin epithelial layer (Kuonen et al. 2013). It is known that, in wound healing processes, many different cell types are involved, including fibroblasts and keratinocytes. As fibroblasts are responsible for initiating angiogenesis, epithelialization, collagen formation and synthesis of extracellular matrix proteins an important step of the proliferative phase of wound healing is the activation of fibroblast migration into the wounded area. An in vitro study showed that *Viscum album* L. liphophilic extract and its predominant triterpene–oleanolic acid significantly and dose-dependently promoted the migration of NIH/3T3 fibroblasts, thereby leading to an enhanced wound closure (Kuonen et al. 2013).

## Antiulcer activity

We found a research regarding the antiulcer activity of mistletoe. Methanolic extract of *Viscum articulatum* Burm. f was tested in Pyrolus ligation ulcer and ethanol induced ulcer models in rats. The extract showed significant inhibition of the gastric lesions in both models. Significant reduction in gastric volume, free acidity and ulcer index was observed compared to control. The authors proposed that antiulcerogenic and ulcer healing properties might be due to antisecretory activity of mistletoe (Naganjaneyulu et al. 2011).

## Antibacterial activity

In the need to find new potent antibacterial compounds, the activity of *Viscum* species was examined in vitro using wide selection of bacterial strains such as *Staphylococcus aureus*, *Staphylococcus epidermidis*, *Bacillus subtilis*, *Bacillus atrophaeus, Enterococcus faecium, Escherichia coli, Bordetella bronchisiptica, Salmonella typhi, Pseudomonas aeruginosa, Pseudomonas syringe, Enterobacter cloacae, Proteus vulgaris, Proteus mirabilis, Klebsiella pneumoniae, Klebsiellaaerogenes, Serratiamarcescens, Streptococcus pyogenes, Mycobacterium tuberculosis, Erwinia carotovora, Agrobacterium tumefaciens, Propionibacterium acnes* and *Xanthomonas campestris* (Satish et al. 1999; Deliorman et al. 2001; Erturk et al. 2003; Oguntoye et al. 2008; Sengul et al. 2009; Hussain et al. 2011; Turker et al. 2012; Assaf et al. 2013; Abualhasan et al. 2014; Kusi et al. 2015; Shah et al. 2017). The results obtained are difficult to compare, because researchers used different solvents and various extortion methods to obtain extracts. In addition, some extracts were obtained from the entire plant, and others were obtained from its individual parts such as fruits, leaves or steams (Hussain et al. 2011; Shah et al. 2017). Mistletoe was also obtained from various types of host trees (Deliorman et al. 2001; Turker et al. 2012). Those interested are invited to read the quoted articles. We found only one in vivo study carried out on rats infected by *Staphylococcus aureus* + *Bacillus cereus, Escherichia coli* + *Pseudomonas aeruginosa, Staphylococcus aureus* + *Pseudomonas aeruginosa* and *Escherichia coli*. Haematological and histopathological studies, after 7 days of treatment with methanolic extract of *Viscum album* L. parasitizing cocoa trees, exhibited its therapeutic effect (Yusuf et al. 2013) (Table 2). Researchers have noticed that antibacterial activity of the extracts was more effective against Gram-negative bacteria than against Gram-positive bacteria (Erturk et al. 2003; Hussain et al. 2011) and postulated that the antibacterial effects were through anti-biofilm activity (Kenar et al. 2016).

## Antifungal activity

Antifungal activity was most often tested on *Candida* species, which are important pathogens causing substantial morbidity and mortality in hospitalized critically ill patients (Jahagirdar et al. 2018). Nacsa-Farkas et al. (2014) tested activity of ethanolic extract of *Viscum album* L. against twelve *Candida* species, of which the most sensitive was *Candida inconspicua* (MIC 5.65 mg/mL). A methanolic extract of *Viscum cruciatum* Sieber ex Boiss. leaves exhibited activity against *Candida albicans* with MIC 1.25 mg/mL (Assaf et al. 2013). Furthermore, an n-hexane extract of *Viscum album* subsp. *abietis* (Wiesb.) Abrom. and its two fractions after flash column preparation were tested against *Candida albicans*. The first fraction was active at a concentration of 1 mg/mL (ZI (zone inhibition) 11 mm), whereas the second fraction showed activity at a concentration of 10 mg/mL (ZI 10 mm) (Erturk et al. 2003). Shah et al. (2017) compared the activity of different parts of *Viscum album* L. against *Candida albicans*. For steam distillations, the most active was the butanol extract, which showed 25.66 and 30.00 mm ZI at 1 and 2 mg/disc, respectively. For leaves, the methanol extract was the most active, reducing the growth of *Candida albicans* as 25.00 and 30.00 mm ZI at 1 and 2 mg/disc, respectively. For fruits, the most potent was the n-hexane extract, which reduced the growth of *Candida albicans* by 22.00 mm ZI at both concentrations (1 and 2 mg/disc). On the other hand, methanolic, dichloromethane and aqueous extracts of *Viscum capense* L. f. stems had no effect on the growth of *Candida albicans* (Amabeoku et al. 1998). Furthermore, Hussain et al. (2011) studied various extracts of *Viscum album* L. leaves and twigs and found that they were not effective against *Saccharomyces cerevisiae* and *Aspergillus flavus*. Methanolic extracts of leaves of *Viscum album* L. growing on cocoa and cola trees inhibited the growth of *Fusarium oxysporium, Penicillium oxalium* and *Microsporum canis*, but the highest activity was obtained against *Aspergillus niger* (ZI 10.66 ± 1.45 mm) (Yusuf et al. 2014). The petroleum ether extract of *Viscum album* L. leaves was found to be more effective against *Aspergillus niger*, *Fusarium oxysporium, Botryodiplodia theobromae* and *Geotrichum candidum* than methanolic extract. The highest activity was exhibited by the petroleum ether extract against *Botryodiplodia theobromae* with ZI of 15.85 mm (Akalazu et al. 2016). It was proposed that two compounds, caprylamide and linolelaidic acid methyl ester, might be the major antifungal compounds in the leaves of mistletoe (***Akalazu et al. 2016). Other compounds active against fungi might be viscotoxines. Viscotoxin A3 and viscotoxin B, at a concentration of 10 µM, blocked the germination of spores, and at lower concentration than 10 µM, inhibited the mycelial growth of *Fusarium solani, Sclerotinia sclerotiorum* and *Phytophthora infestans*. It was postulated that viscotoxin A3 might bind to membranes and form ion channels, leading to destabilization and disruption of the plasma membrane (Giudici et al. 2003, 2004, 2006).

## Antiviral activity

So far, the antiviral activity of mistletoe has not been extensively investigated. The aqueous extract of leaves of *Viscum album* L. growing on lime trees was found to be effective against human parainfluenza virus type 2 (HPIV-2) growth in Vero cells. The extract, at a dose of 1 µg/mL, inhibited HPIV-2 replication and suppressed virus production by 99.7% (ED50 0.53 µg/mL) with no toxic effect on host cells (Karagöz et al. 2003). The methanolic extract of *Viscum album* L. leaves was active against measles virus growth in Vero cells at 0.063 μg/*μ*L (IC_50_ 0.031 μg/*μ*L) and 0.031 μg/*μ*L (IC_50_ 0.039 μg/*μ*L). On the other hand, the polio, yellow fever and simplex virus-1 (HSV-1) viruses were resistant to the same extract (Obi and Shenge 2018). Due to the strong impact of mistletoe on the immune system, it was proposed that it might be useful as a complementary treatment of patients with human immunodeficiency virus (HIV). Studies to confirm the induction of immunomodulation and possibility of the inhibition of the progression of HIV disease are needed (Gorter et al. 1996, 1999; Stoss and Gorter 1998; Stoss et al. 1999). An in vitro study revealed that a phenolic glycoside, homoeriodictyol-7-*O*-*β*-D-glucopyranoside-4′-*O*-*β*-D-apiofuranoside, isolated from *Viscum articulatum* Burm. f. growing on Fagaceae: *Lithocarpus variolosus* (Franch.) Chunex showed weak anti-HIV-1 activity with CC_50_ > 200 μg/mL and EC_50_ = 18.09 μg/mL. This compound exerted its weak protection of HIV-1ШB-induced MT-4 host cell lytic effects with a TI > 11.06 (Li et al. 2008).

## Conclusion

Traditionally mistletoe has been used in a treatment of many diseases. To date, the anticancer and immunomodulatory activities of *Viscum* species were the most studied. In Europe, mainly in German-speaking countries, this resulted in the launch of extracts for subcutaneous or intravenous administration to improve the quality of life and survival of cancer patients. Lately, an extensive number of in vitro and in vivo studies have been conducted to investigate the other pharmaceutical activities of mistletoe. The results of these studies (Table2) showed that mistletoe might be a potential source of new drugs and complementary therapies to treat hypertension, diabetes, liver diseases, epilepsy and Alzheimer’s disease. Furthermore, it might be used to improve endurance and muscle strength, enhance wound healing and as antibacterial and antifungal agent. Such a wide variety of pharmaceutical properties is due to the content of many biologically active compounds from various chemical groups. The chemical composition of mistletoe depends on part of the plant (stem, leaves, fruits) and host species as well as the place and time of harvest. To date, the active compounds responsible for the individual pharmacological activities of mistletoe have not been identified. Further studies on fractionation and isolation of main active compounds and the development of methods of standardization of the extracts are required. Such studies should be conducted for extracts prepared from mistletoe parasitizing various host trees, different harvesting periods and different parts of the plant. In the next step of research, the mechanisms of action need to be tested, not only for individual isolated active compounds, but also for the whole extracts. This is because the therapeutic effect of mistletoe might be a result of the synergistic interactions of various secondary metabolites. Those interactions might include both low-molecular weight compounds (such as phenolic acids, flavonoids and fatty acids) and high-molecular weight substances (such as viscotoxines and lectins). Because of the diverse synergistic interactions, the mechanism of action of mistletoe might include many signalling pathways. Mistletoe might regulate either the same or different targets in various pathways, while acting on membrane receptors, enzymes, ion channels, transporter proteins and transcriptional targets. In this review, we summarized the existing studies on pharmacological activities of *Viscum* species and proposed possible mechanisms of action. Still, this is a new field for scientific research, and further studies on compound isolation and identification, synergistic interactions, metabolism, mechanisms of action and toxicity are required. We believe that due to this research, mistletoe will become a source of new complementary therapies supporting the treatment of many diseases.
